# Unveiling the Molecular Footprint: Proteome-Based Biomarkers for Alzheimer’s Disease

**DOI:** 10.3390/proteomes11040033

**Published:** 2023-10-16

**Authors:** Mukul Jain, Rupal Dhariwal, Nil Patil, Sandhya Ojha, Reshma Tendulkar, Mugdha Tendulkar, Parmdeep Singh Dhanda, Alpa Yadav, Prashant Kaushik

**Affiliations:** 1 Cell and Developmental Biology Laboratory, Research and Development Cell, Parul University, Vadodara 391760, India; rupal123dhariwal@gmail.com (R.D.); patilnil910@gmail.com (N.P.); 2 Department of Life Sciences, Parul Institute of Applied Sciences, Parul University, Vadodara 391760, India; san15720@gmail.com; 3 Vivekanand Education Society, College of Pharmacy, Chembur, Mumbai 400071, India; reshma.tendulkar@ves.ac.in; 4 Sardar Vallabhbhai Patel College of Science, Mira Rd (East), Thane 400071, India; mugdhatendulkar14@gmail.com; 5 Department of Biochemistry, Punjab Agricultural University, Ludhiana 141027, India; parmdeepdhanda@gmail.com; 6 Department of Botany, Indira Gandhi University, Meerpur, Rewari 122502, India; alpa.yadav21@gmail.com; 7 Instituto de Conservacióny Mejora de la Agrodiversidad Valenciana, Universitat Politècnica de València, 46022 Valencia, Spain

**Keywords:** Alzheimer’s disease, biomarkers, proteomics, microarray, bioinformatics

## Abstract

Alzheimer’s disease (AD) is a devastating neurodegenerative disorder characterized by progressive cognitive decline and memory loss. Early and accurate diagnosis of AD is crucial for implementing timely interventions and developing effective therapeutic strategies. Proteome-based biomarkers have emerged as promising tools for AD diagnosis and prognosis due to their ability to reflect disease-specific molecular alterations. There is of great significance for biomarkers in AD diagnosis and management. It emphasizes the limitations of existing diagnostic approaches and the need for reliable and accessible biomarkers. Proteomics, a field that comprehensively analyzes the entire protein complement of cells, tissues, or bio fluids, is presented as a powerful tool for identifying AD biomarkers. There is a diverse range of proteomic approaches employed in AD research, including mass spectrometry, two-dimensional gel electrophoresis, and protein microarrays. The challenges associated with identifying reliable biomarkers, such as sample heterogeneity and the dynamic nature of the disease. There are well-known proteins implicated in AD pathogenesis, such as amyloid-beta peptides, tau protein, Apo lipoprotein E, and clusterin, as well as inflammatory markers and complement proteins. Validation and clinical utility of proteome-based biomarkers are addressing the challenges involved in validation studies and the diagnostic accuracy of these biomarkers. There is great potential in monitoring disease progression and response to treatment, thereby aiding in personalized medicine approaches for AD patients. There is a great role for bioinformatics and data analysis in proteomics for AD biomarker research and the importance of data preprocessing, statistical analysis, pathway analysis, and integration of multi-omics data for a comprehensive understanding of AD pathophysiology. In conclusion, proteome-based biomarkers hold great promise in the field of AD research. They provide valuable insights into disease mechanisms, aid in early diagnosis, and facilitate personalized treatment strategies. However, further research and validation studies are necessary to harness the full potential of proteome-based biomarkers in clinical practice.

## 1. Introduction

The most common factor for dementia is Alzheimer’s disease; it is estimated that over 50 million people suffer from the disease worldwide. The healthcare industry exerts massive efforts to treat patients by modifying therapeutics. A large number of AD cases arrive in the later stages of life or after 65 years, and only 5% of cases are of early-onset, while 1–2% occur because of family history. The cure for this devastating ailment is symptomatic because the precise origin of the brain’s degeneration is unknown and its pathogenesis is harmonious with genetic or genomic, and biochemical or proteomic causes [1].

### 1.1. Importance of Biomarkers in AD Diagnosis and Management

Biomarkers or biological markers are bio-molecules or indicators that can be measured, analyzed, and evaluated so that the pathogenic process or pathogenic processes can be easily identified, and they can also help evaluate the pharmacological response towards therapeutic intervention. Biomarkers are used as an indicators of good health and disease. Biomarkers are specific, sensitive, and can be utilized easily to define the stages of the disease, which makes them useful for diagnostic criteria [2]. Over the years, most research has focused on finding the connections between pathological biomarkers, like neurofibrillary tangles, cortical amyloid plaques, and mutations associated with protein components [3], and the tau protein—a tubule-binding protein [4]. AD is a complex syndrome, in which biomarkers support early diagnosis and prognostic assessment, to relate to different stages of disease, which could help researchers to understand the mechanism of the disease and to understand clinical responses [5]. The new guideline of the Institute on Aging and Alzheimer’s Association says that AD terminology is used when biological biomarkers are present, e.g., NFT and Aβ aggregates. Biomarkers are classified according to the way they are identified, such as “A” biomarkers, which are amyloid aggregates, tested by amyloid positron emission tomography [PET]; Aβ42 and Aβ42/40 ratio-tested biomarkers from cerebrospinal fluid [CSF]; “T” refers to PET Tau and p-Tau CSF; and “N” represents magnetic resonance imaging, neurofilament light chain protein [NFL], CSF total Tau [t-Tau], and fluorodeoxyglucose [FDG] [6].

### 1.2. Role of Proteomic Identifying Biomarkers

Proteomic biomarkers are disease-related biomolecules [proteins] that can help to diagnose and monitor the stages of the disease and suggest the molecules that can be targeted for treatment or can be evaluated for therapeutic responses [7]. As the disease starts, it changes the protein expressions in tissues and biofluids of the body, which indicates the diseased condition and its early detection. The proteomic technique, used in molecular medicine and biomarker discovery, is carried out by protein profiling of body fluid. Proteomics gives valuable disease-specific biomarkers identification through normal and diseased conditions. The simplest approach to identifying proteomic biomarkers is 2D-PAGE [8]. This proteomic biomarker can be classified into three categories: prognostic, diagnostic, and treatment predictive, according to the information provided by them [9]. A prognostic biomarker can predict the occurrence of disease in the future, a diagnostic biomarker helps to identify the disease, and a predictive biomarker is used as a tool to design the drug [10]. Proteomics is useful for identifying the proteins that are involved in the progression of the disease, after the identification of biomarkers by mass-spectrometry, then biomarkers need to be processed using bioinformatic analysis and would be reproduced in different populations [11]. Proteomic techniques are promising tools in the detection of biomarkers from biofluids like urine, blood, and serum because they are less invasive and are cost-effective [12]. In the last few years, MS has expanded its role in proteomic studies, in almost all fields of science. Drug discovery is a very complex process and has a high cost, but proteomics accelerates and facilitates the process of drug discovery. It also plays a major role in the identification of toxicity, resistance, target identification steps, and efficacy [13]. Proteomics is considered for cell protein analysis, to understand the pathways or cycle, and the role of the drug that can counteract or inhibit the disease-associated biochemical processes, the network of protein interaction, and the cellular-level mechanism, during clinical trials [14].

## 2. Alzheimer’s Disease: Pathophysiology and Proteomic Approaches

The Amyloid-beta and Tau proteins are both soluble tissue components in a normal state when denatured, by some events, when a disease arrives that is not directly related to the overproduction of these physiological hallmarks. These facts were confirmed by Alois Alzheimer after the description of the signature epiphenomenon of AD, showing that tangles and plaques are not the only cause of neurodegeneration [15]. Researchers are trying to find relationships between the aggregation of the proteins, synaptic disintegration, and neuronal loss, which is unproven till now, and favors the amyloid cascade hypothesis [16].

### 2.1. Overview of AD: Pathology and Molecular Mechanism

The early stages of clinical manifestation include amnesia, progressive memory loss, cognitive decline, and some severe pathological changes, such as degeneration of cortical matter and the hippocampus [17]. As well as these characteristics, a few additional comorbidities are also manifested like psychological disorders, movement problems, and sleep disturbances [18]. Investigation of FAD (dominantly inherited familial AD) reveals mutation of the APP (Amyloid-Precursor Protein) gene and PSEN (Presenilin) that cleaves the protein and forms Aβ from APP, and tau is responsible for frontotemporal dementia [19], which supports another hypothesis apart from toxic protein aggregate formation that helps us to understand the neurodegeneration mechanism [20]. Molecular studies have discovered that amyloid plaques contain amyloid-beta peptide and NFT comprising hyperphosphorylated tau, which helps us to classify the disease [21]. In 1984, for the first time, Aβ peptides were isolated by Wong and Glenner from AD patients [22], therefore the sequencing of APP (Amyloid-Precursor Protein) became possible [23], which leads to the subsequent cloning of the protein [24]. This biochemical analysis has driven new ideas towards genetic mapping of mutations in the causative genes of AD, inclusive APP [in 1991] and Presenilin 1/2 [25]. Analysis of the genetic and biochemical evidence concludes that consecutive proteolysis of APP (Amyloid-Precursor Protein) by BACE1 and gamma-secretase [PSEN1/2 are part of the gamma–secretase complex] is responsible for amyloidogenic Aβ peptide formation [26]. In 1986, the dominant component of NFT, hyperphosphorylated Tau, was purified from the brain tissue of an AD patient [27], helping to explain the mutation in the Tau gene and its critical role in the progression of AD [28], and establishing the Tau hypothesis. Further studies suggest that amyloid aggregates initiate cognitive defects and the downstream of the Tau protein drives the neurotoxicity that causes neuronal death [29]. Other pathological consequences play a key role in AD, including synaptic dysfunction, vascular dysregulation, and inflammation. The diagnostic criteria depend on clinical data and biological definition is based on the development of biomarkers that consider whether neuropathology is required. AD is a complex syndrome, in which biomarkers support early diagnosis and prognostic assessment that relate to different stages of the disease, which could help us to understand the mechanism of the disease and to understand clinical responses [30]. The list of other proteins and biomarkers implicated in AD is provided in Table 1.

### 2.2. Introduction to Proteomics and Its Application in AD Research

National institutes on aging have initiated some steps that strengthen the Accelerating Medicines Partnership [AMP]-AD, a multidisciplinary strategic program between industry and academia, their aim is to discover novel targets of biomarkers as well as their therapeutic partners [32]. This new approach offers an indispensable tool to better understand the complexity of AD by multi-omics analysis. While other fields like the genetics of AD have been evaluated extensively, in this review we trying to review the proteomics of AD. In recent years, researchers have focused on deep brain proteome profiling [33], dissecting the sub-proteome, analyzing large samples, evaluating complex PTM motifs, and identifying biofluid biomarkers [34].

### 2.3. Challenges in Identifying Reliable Biomarkers

The analytical techniques of biomarkers are already well established, however, some issues are still unaddressed and need proper interpretation and unique clinical diagnostic support [35]. The collection of samples is equally important for biomarker stability and quality, and the mishandling of a sample can destroy biomarkers. Storage, transportation, and initial processing are equally important for saving biomarkers in the sample because these are biomolecules (DNA, RNA, and proteins) that can be easily denatured. Analytical methods and techniques are expensive and invasive, therefore cheaper and non-invasive methods are required for the identification of biomarkers, and biomarkers should have longer shelf-lives that provide stability and accuracy during analysis. Tests should be performed in triplet so the test accuracy increases by 68%, which can be affordable only for 10% of patients. There are limitations to the testing of protein biomarkers, which are lowly and highly expressed proteins in the same well. Low-level proteins need 1:2 dilutions to be expressed, but high-level proteins can be expressed in lower dilution, so both cannot be quantified in the same well, which increases the cost of analysis and data interpretation, which is equally important to promoting clinical diagnosis [36]. The collection of healthy controls is crucial for proteomic biomarker research. Finding individuals with matching characteristics like sex, education, and lifestyle is exceptionally challenging, and data validation is essential for scientific credibility, requiring support from others.

## 3. Proteomics Techniques for Biomarker Discovery in Alzheimer’s Disease

Alzheimer’s disease, a devastating neurodegenerative disorder, poses a significant challenge for early diagnosis and treatment. In the quest to uncover biomarkers that could aid in early detection, proteomics techniques have emerged as powerful tools with immense potential. Some tools are described below:

### 3.1. Gel-Based Quantitative Technique and Differential Proteomics

Gel electrophoresis serves as a laboratory method employed to segregate and examine macromolecules, relying on their size and charge differentials by implementing an electric field through a gel matrix [37]. Widely applied in molecular biology and genetics, this technique facilitates the analysis of DNA, RNA, and proteins. In the realm of proteomics, it plays a pivotal role in identifying biomarkers, mainly concentrating on two variants: 2D PAGE and SDS-PAGE. The 2D-PAGE method combines two separation dimensions for higher resolution. It involves separating proteins from biological samples based on their isoelectric point and size using polyacrylamide gel electrophoresis [38]. Within the realm of neuroproteomics, conventional investigations have often utilized 2-DE and 2D-DIGE as the primary methods for comparing protein patterns across varying scenarios [39]. Over the years, multiple methods have emerged to expand and improve 2D-PAGE, resulting in a reliable and consistent approach. The implementation of immobilized pH gradient (IPG) strips, a replacement for tube gels with ampholytes, has successfully eradicated the problem of ‘cathodic drift’ during isoelectric focusing (IEF). This advancement has led to a notable increase in the reproducibility of samples, making the technique more reliable for proteomic studies. The development of narrow pH ranges in IPG strips (e.g., 4–7, 5–8) enables better separation of proteins with similar isoelectric points than traditional broad pH range strips (e.g., 3–10) [40]. However, solubilization remains a challenge in proteomics since ionic detergents like SDS (used in SDS-PAGE) cannot be used for lipidated proteins, like transmembrane proteins, due to interference with the focusing process in IEF [41]. SDS has been used in some cases, but dialysis of samples before IEF is necessary. However, this poses a limitation when working with precious biological samples due to potential sample loss. In response to this challenge, chaotropic agents like urea and thiourea, in conjunction with zwitterion detergents (e.g., CHAPS) have been utilized to avoid protein precipitation during both IEF and SDS-PAGE [42]. Tributyl phosphine has been used as a reducing agent instead of dithiothreitol [43]. Despite advancements in 2D-PAGE techniques, challenges and limitations remain. Solubilizing methods are predominantly constrained to cytosolic proteins, posing difficulties in generating gel maps for membranous and lipidated proteins. Quantification of protein alterations relies on 2D image analysis software, often demanding replicates for comparison and spot alignment. To address inter-gel disparities, 2D-DIGE employs fluorescent cyanine dyes (Cy2–Cy5) for distinct sample labeling, enabling their consolidation and simultaneous electrophoresis in a single 2D gel [44]. Within the context of 2D-DIGE, it becomes possible to quantify individual spots on a single gel, and the alignment of multiple gels can be achieved through referencing an internal standard labeled with Cy2 [45]. According to Naseri’s report in 2020, 2D-DIGE identified two client proteins, SNAP-25, and dynamin-1, suggesting that abnormal protein palmitoylation may play a crucial role in the development of ND [46].

### 3.2. Mass Spectrometry (MS) for Protein Identification and Quantification

**Figure 1:**
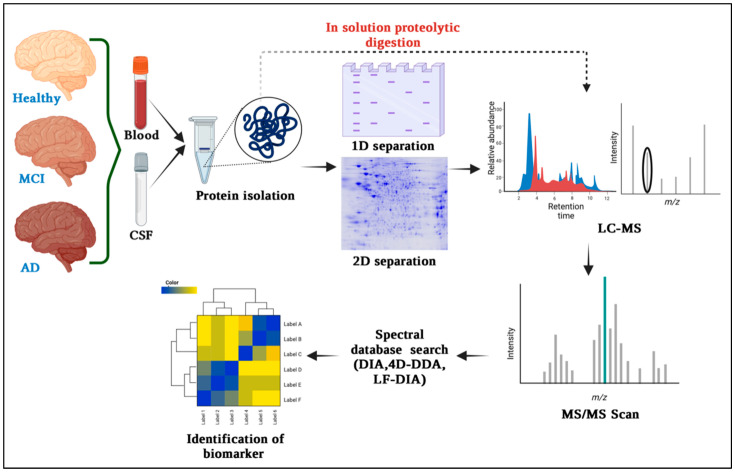
Overview of mass spectrometry-based proteomics techniques commonly used for biomarker discovery in Alzheimer’s disease. It includes protein separation, peptide identification, and quantification steps, which contribute to the identification of potential biomarkers.

In recent years, there has been a notable shift from 2D gel-based approaches to MS-based neuroproteomics studies [47]. The shift is primarily steered by progress in MS instrument design, the integration of resilient quantitative MS methods, adaptation for working with limited protein quantities in neuroproteomics research, and the increasing fascination with exploring complex membrane proteins that pose challenges for analysis through traditional 2D gel techniques [48]. Even as MS-based investigations employing stable isotope labels or label-free methodologies gain traction, gel-based techniques remain prevalent owing to their accessibility and straightforward preparation, particularly when dealing with a restricted number of samples. After detecting protein spots of interest, the proteins must be eluted from the gel for MS analysis. Usually, the spots corresponding to the desired protein(s) are apart from the gel and undergo diverse treatments and chemical alterations to aid protein fragmentation by a protease, resulting in the formation of multiple peptides [49]. The process of peptide mass fingerprinting involves obtaining characteristic mass fingerprints of a protein by analyzing the smaller peptides resulting from protease cleavage, and their molecular weights are determined using MS Figure 1 [50]. In this method, MS analysis is used to determine the experimental masses. Subsequently, a database search is conducted where the experimental masses are compared to in silico ‘digestion’ generating protein-specific mass fingerprints. The identification of the protein of interest is determined based on the quality of peptide matches. Mass spectrometry is indispensable for protein identification and proteomic analysis. Before the development of ‘softer’ ionization techniques, protein identification relied on specific antibodies or Edman degradation and protein sequencing, which required educated guesses based on molecular weight and pI knowledge [51]. Edman degradation and database searching are lengthy and labor-intensive methods. The two most common techniques for MS analysis of proteins are matrix-assisted laser desorption ionization (MALDI) and electrospray ionization (ESI) [52]. MALDI involves ionizing proteins using a laser in a time-of-flight mass spectrometer, providing precise mass-to-charge ratio determination. It offers high-throughput capabilities, simplifies sample preparation, and allows direct analysis of intact proteins and peptides from complex samples. Comparing mass spectra from control and disease samples helps identify differentially expressed proteins, making MALDI-TOF a pivotal tool for biomarker discovery and disease understanding. MALDI-TOF MS is highly valuable for peptide mass fingerprinting (PMF), a well-established protein identification method [53]. PMF entails the comparison of a distinctive list of peptide masses generated by targeted protein cleavage with a computer-simulated list of peptides resulting from the digestion of established protein sequences stored in databases [54]. To confirm protein identification, tandem spectrometry (MS/MS) can be used with various search engines (e.g., Mascot, ProFound, Sonar, MS-Fit, Sequest) that offer simple, fast, and automated protein identification (MS and/or MS/MS search). Nonetheless, it is crucial to take into account that the acquired outcomes are statistical in nature and to be mindful of the constraints inherent in the statistical identification approach. As noted by [55], blood emerges as an appealing avenue for biomarker exploration due to its ease of access and the existence of proteins originating not solely from blood but also from other tissues, courtesy of its systemic circulation within the body [56]. Blood-found proteins provide valuable insights into the organism’s health status. With MALDI-TOF MS, there is no need for pre-selecting biomarker candidates as multiple compounds can be analyzed in one experiment, offering a significant advantage [57]. For slowly progressing disorders like AD, identifying and monitoring biomarkers is crucial for evaluating the efficacy of disease-modifying drugs. Biomarker study data from clinical trials inform decisions on drug progression. Researchers investigate strategies such as γ-secretase inhibitors against βA and cholinergic system therapies. Portelius et al. used MALDI-TOF MS with immunoprecipitation, revealing that shorter βA isoforms (e.g., βA (1–14), βA (1–15)) are more γ-secretase inhibitor-responsive than longer ones (e.g., βA (1–40) or βA (1–42)) [58]. The combination of MS and immunoprecipitation for βA analysis proves essential in targeted βA proteomics. MS enables accurate identification and verification of proteins and antigens. In 1993, the first targeted βA proteomics using MALDI-TOF MS detected multiple βA isoforms in CSF. New findings from MALDI-TOF MS studies have exposed an alternative pathway for APP (Amyloid Precursor Protein) cleavage, with α- and β-secretase playing a role distinct from γ-secretase [59].

### 3.3. Protein Microarrays and Antibody-Based Techniques

Protein microarrays are a miniaturized version of traditional biochemical assays, where hundreds to thousands of different proteins or peptides are immobilize in a spatially addressable manner on a solid substrate, such as a glass slide or a silicon chip [60]. These immobilized proteins can be used to study various aspects of protein function, including protein–protein interactions, protein–ligand interactions, protein-DNA interactions, enzyme activities, and post-translational modifications. In a specific investigation, scientists employed cDNA microarrays containing 18,000 genes to scrutinize cDNA samples from hippocampal CA1 neurons. These samples were obtained from Alzheimer’s patients with and without neurofibrillary tangles, along with control subjects. Similarly, prefrontal cortex samples from individuals with schizophrenia and controls were screened using 7000-gene arrays to detect gene expression variations. This screening unveiled decreased expression of genes regulating presynaptic function. Validation of the observed gene expression changes via methods like immunohistochemistry, in situ hybridization or reverse transcription polymerase chain reaction is crucial [61]. Another illustrative case pertains to the utilization of microarrays to examine the transcriptional profile of brain plaques from multiple sclerosis (MS) patients, juxtaposed with control brain samples. This study effectively identified the exclusive presence of osteopontin (OPN) gene expression within MS plaques. As a result, it was proposed that this pro-inflammatory molecule is generated by infiltrating T lymphocytes, microglia, and macrophages, contributing to myelin sheath damage via an autoimmune mechanism. Furthermore, it is evident that alterations in the OPN gene might affect disease progression [62]. In Ho et al.’s 2005 study, 6794 human genes were screened, revealing 32 aberrantly expressed genes (25 known and 7 unknown, based on EST) in the superior temporal gyrus of moderate dementia cases compared to cognitively normal controls (>1.8-fold difference) [63]. These findings highlight the potential significance of these genes in early stage AD. Further research is needed to elucidate their precise role(s) in AD development and progression. In Kim JR et al.’s 2003 study, microarray experiments examined gene dysregulation in AD animal models. Introducing pathogenic mutations of APP, presenilin, and tau in mice led to AD pathologies like amyloid plaques and neurofibrillary tangles [64]. Expression profiling identified the downstream effects of these mutations in transgenic AD animal models. In conclusion, microarray-based techniques have emerged as powerful tools for proteome-based analysis in Alzheimer’s disease (AD). They allow for the comprehensive profiling of protein expression levels and can help identify key biomarkers associated with AD pathology; unlocking the potential of Western blot analysis, an independent technique crucial for studying protein expression. Through gel electrophoresis, it separates proteins based on size and charge, followed by antibody binding to the target protein on a membrane. Detection methods then reveal vital information about the protein’s presence and abundance, making it invaluable for AD research. Antibody selection for respective Aβ recognition motifs is a crucial part of Western blotting. Both monoclonal and polyclonal types of antibodies are used in Western blotting. Some of the major antibodies are described in Table 2 below.

Pryor et al. employed the A8 monoclonal antibody, designed to target oligomers, as a substitute for the preparation of Aβ1–42. The antibody A8 displayed a range of oligomer species with sizes spanning from 16.5 to 25 kDa [80]. In contrast, the 6E10 antibody provided poorer resolution, displaying larger species of oligomers. These findings imply that 6E10 might exhibit a more potent response to oligomers with greater molecular weight, or alternatively, the antibodies might exhibit a preference for binding to various sizes of Aβ1–42 oligomers [81]. While Western blotting assists in identifying intermediate Aβ oligomers, the prevalent gel smear in numerous studies suggests its inability to precisely quantify individual oligomer sizes in this range [82,83]. Interestingly, a recent study investigated a comparison between age-matched control cases exhibiting normal cognitive status (CDR 0) and individuals with mild cognitive impairment (MCI) (CDR 0.5). The researchers observed a significant N2-fold reduction in splice variants I–III of the a-type synapsin isoform within the entorhinal cortex of MCI cases [84]. Interestingly, there were no significant alterations noted in splice variant II of the b-type synapsin isoform or in synaptophysin within the same EC region. Notably, the modified expressions of synapsin a-type isoforms were exclusive to the EC in cases of MCI, as no observable decreases were found in the VC of the same individuals [85]. These groundbreaking discoveries illuminate the importance of selectively modified gene expression in the initial detectable phase of AD dementia. They offer valuable molecular evidence.

### 3.4. Advancements in High-Throughput Proteomics Technologies

Some of the high throughput proteomics technologies such as TMT (tandem mass tag), cysteine-reactive tandem mass tag (cysTMT), OxcysDML, isobaric tags for relative and absolute quantitation (iTRAQ), quantitative thiol reactivity profiling (QTRP), and electrophilic diazene probe (DiaAlk) were used for proteomics analysis in AD and other ND. In the work proposed by Mei Chen et al., 2020, TMT (tandem mass tag) was used for proteomic study of body fluids from AD patients. This technique enables relative quantitation of proteins present in multiple samples by labeling peptides with stable isotope tags that fragment into reporter-ions upon collision-induced dissociation [86]. Also, redox proteomics can detect hundreds to thousands of oxidized proteins in a single experiment and this is attractive for understanding the redox status of proteins. Thus, in another work in 2014, Garcia-Santamarina et al. proposed the OxcysDML method for quantifying cysteine redox in a demonstration capacity. This technique was employed to examine the liver proteome of a mouse model with Alzheimer’s disease, with the goal of enhancing comprehension regarding redox chemistry in the condition. As per their results, nearly 90% of cysteine was observed in its reduced state within the living organism. Given the prominence of cysteine in the mouse proteome (accounting for around 14% of all in silico tryptic peptides containing cysteine), the scientists approximated that only about 2% of tryptic peptides (equivalent to roughly 4 μg per sample) underwent enrichment and were subsequently subjected to analysis using OxcysDML [87]. In 2008, Bronwen Martin conducted a study where they demonstrated the use of the iTRAQ control protocol alongside 3xTgAD tissue samples. Concurrently, both the control and AD samples underwent treatment throughout the labeling process. This labeling protocol encompassed multiple stages: protein reduction and cysteine blocking, protein trypsin digestion, peptide labeling using iTRAQ reagents, merging the samples for comparison, employing strong cation exchange chromatography, conducting solid phase extraction for desalination, and concluding with LC/MS/MS analysis [88]. Through this comprehensive approach, they were able to unravel some of the intricate proteome changes that occur in a mouse model of Alzheimer’s disease. These discoveries hold the promise of unveiling fresh therapeutic avenues for Alzheimer’s disease (AD) and other neurodegenerative disorders (ND). Progress in high-throughput proteomics technologies has transformed the realm of proteomics, empowering scientists to examine extensive protein samples rapidly and efficiently, marking a revolutionary shift in the field. Through innovative techniques, such as mass spectrometry-based approaches and quantitative proteomics, scientists can now gain deeper insights into complex biological systems, paving the way for groundbreaking discoveries in disease mechanisms, drug development, and personalized medicine [89]. As these technologies continue to evolve, the future holds great promise for unraveling the intricacies of the proteome and its vital role in various physiological and pathological processes.

## 4. Candidate Proteome-Based Biomarkers for Alzheimer’s Disease

### 4.1. Amyloid-Beta (Aβ) Peptides and Tau Protein

Current disease models show that Amyloid-beta in either non-fibrillary, soluble, oligomer, or plaque form initiates tau misfolding and assembly through a pathophysiological cascade that helps in its spread throughout the cortex, causing neuronal system failure, neurodegeneration, and cognitive decline [90]. The amyloid precursor protein (APP) and Presenilin mutations cause the accumulation of pathological Aβ species in the brain resulting in early-onset AD pathogenesis [91]. Normally, the beta and gamma-secretase enzymes generate soluble amyloid-beta fragments by cleaving the APP protein. However, a mutated condition in the APP gene forms insoluble Aβ fragments that eventually convert into clumps. These toxic Aβ species manipulate the normal tau phosphorylation regulating the function of protein kinases and phosphatases, inducing tau misfolding, and tangle formation [92]. Aβ pathogenicity requires tau toxicity as the tau mediates synaptic dysfunctioning and neuronal cell death, thereby enhancing memory deterioration and cognitive impairment in AD [93]. It is hypothesized that amyloid beta generation is initiated during the early stages of Alzheimer’s disease, and eventually uprises forming plaque deposits that progressively increase in size and downregulate glutamatergic transmission and damage the associated synapses [94]. A network dysfunction is generated at the site, close to deteriorated synapses where the microglial cells exhibit a response to remove the damaged synapses and prevent further damage. However, as the amyloid-β plaque deposition spreads across multiple regions in the brain, the synaptic damage becomes more prominent and spreads, causing tau hyperphosphorylation, tau dissociation from microtubules, and the tau entangle formation, which promotes axon loss and neurodegeneration [95]. These changes in normal brain activity and functioning link with memory loss, cognitive impairments, and brain connectivity dysregulation in a stage-dependent manner, suggesting they may be useful for tracking disease progression. Yan Li et al., 2022, established the discovery of probable amyloid-beta plaques from the CSFAβ_42/40_ ratio, suggesting their potential as a biomarker for AD detection [96]. Hansson et al., 2019, also focused on determining the effect of pre-analytical handling of biomarkers of AD and the quantity retrieved [97]. Similarly, Lih-Fen Lue et al., 2017, emphasized finding these Amyloid-beta and tau proteins in the blood. However, in clinical practice, CSF biomarker analysis involves sampling from patients with atypical or mixed presentation of dementia, making the diagnosis complex, thereby highlighting the importance of AD discrimination from other neurodegenerative processes [98].

### 4.2. Apolipoprotein E (APOE)

Apolipoprotein or ApoE, a 34 kDa glycoprotein with a 299 amino acid long polypeptide chain is a blood–brain barrier (BBB) impermeable protein, present in significant amounts in the central nervous system due to expression of astrocytes, microglia, vascular mural cells, choroid plexus cells [99]. In the CNS, ApoE plays a prominent role in axonal growth and synapse formation, which are crucial for learning, memory generation, and neuronal repair by delivering cholesterol to nerve cells. It is associated with the low-density lipoprotein receptor (LDLR), and LDLR-related protein 1 (LRP1) so as to maintain lipid homeostasis via lipid transport from one tissue or cell type to another [100]. Three varied *APOE* alleles exist in the human body, namely: the ε2 (*APOE2*), ε3 (*APOE3*), and ε4 (*APOE4*). They are distinctive from each other by the varying cysteine and arginine amino acids at the positions 112 and 158 (apoE2: Cys112/Cys158; apoE3: Cys112/Arg158; apoE4: Arg112/Arg158) and they contrastingly regulate the cholesterol levels for γ-secretase activity and Aβ production [101]. Genome-wide association studies deduced ε4 allele polymorphism of APOE as a significant genetic risk factor that deposits with Aβ in amyloid plaques causing late-onset AD [102]. The apoE4 hinders the LRP1 (low-density lipoprotein receptor-related protein 1) receptor-mediated Aβ clearance as it weakly associates with Aβ causing hindrance in the uptake of Aβ/ApoE complexes in neurons [103]. In a normalized state, neuronal apoE4 promotes tau phosphorylation and cell death by modulating microglial activation, however, impaired apoE4 dysregulates homeostatic microglial functioning playing a role in amyloid plaque degradation due to its reduced affinity to TREM2 (triggering receptor expressed on myeloid cells 2) receptors expressed by microglial cells [104]. Recent research demonstrated that ApoE4, independent of Aβ, elicits an inflammatory pathway causing neurovascular dysfunction, including blood–brain barrier collapse, leakage of blood-derived toxic proteins into the brain, and shortened small vessel length [105]. Thus, obtaining *APOE* genotype status has been commended as a necessity for AD therapy as it is a determining factor of AD risk exerting influence on multiple disease pathways [106]. Ying et al., 2021 focus on the elevated CSF ApoE association with longitudinal changes in AD biomarkers including Amyloid-beta and others [107]. Other work by Matthew Paul et al., 2022, focused on finding the imbalance between the different glycoforms of ApoE monomers in AD that cause hindrance with its biological function, contributing to the progression of the disease [108]. However, the presence of a lower amount of APOE4 in CSF, along with a limited sample volume of CSF, lowers the sensitivity of APOE4 detection assays. Thus, for future inventory purposes and drug development for AD, studies need to explore the therapeutic tools for analyzing and modifying certain parameters of ApoE, such as its structure and homeostasis maintaining property, thereby producing changes in pathological AD progression.

### 4.3. Clusterin (CLU) and Other Chaperone Proteins

Alzheimer’s disease (AD) is a persistent and distressing neurological condition affecting older-age populations more frequently. Some proteins, such as clusterin (CLU) or apolipoprotein J (APOJ), have been found to be associated with dementia, neurological inflammation, and oxidative stress during such AD conditions [109]. Clusterin, encoded by the CLU gene located on the p21-p12 locus on chromosome 8 of humans, is an omnipresent and obstinately produced protein renowned as a molecular chaperone expressed by a variety of tissues and body fluids [110]. It is the third-most important genetic risk element for late-onset AD, with a number of variants. The interaction and binding properties of clusterin with Aβ appear to influence aggregation and enhance Aβ clearance, hinting toward the neuroprotective effect [111]. Clusterin inhibits aggregation and helps LRP2 (megalin) in removing Aβ. Tau pathology spreads from cell to cell in a manner similar to prion disease that also may be regulated by extracellular chaperones like Clusterin [112]. Using CLU-deficient animals as models of amyloidosis, the relationship between CLU and Aβ may be discovered in vivo [113]. In contrast to controls, people with Alzheimer’s disease (AD) have higher levels of the mRNA, or messenger ribonucleic acid, of CLU in various parts of the cerebral tissue (brain), according to a study. Further, higher amounts of CLU proteins were also observed in both the hippocampus and frontal cortical regions of post-mortem AD brains [114]. A study using SH-SY5Y cells exposed to AD patients’ CSF shows the cytoprotective ability of Clusterin alone, and also in combination with extracellular chaperones it preserved and protected the cells from damage [115]. Under physiological circumstances, CLU reduces aggregates and mediates Aβ clearance. The co-culture studies on rat hippocampus astrocytes and neurons revealed that Clusterin incubation reduces Aβ-induced astrocytic calcium intake, resulting in diminished ROS formation and caspase 3 activations [116]. A more recent experiment using APP/PS1-mutated mice revealed that Clusterin knockout increases amyloid angiopathy while reducing bleeding and inflammation by shifting Aβ deposition from plaque to deposit in the cerebrovascular fluid. In a cellular model, Clusterin inhibited the development of Tau fibrils but promoted the formation of Tau oligomers to initiate the aggregation of endogenous Tau. Pre-aggregated Aβ was incubated with Clusterin, and this reduced the amount of amyloid that human primary astrocyte cultures and microglia ingested from preparations of fibrils and oligomers [117]. However, this transporter, CLU, is also operative at the blood–brain barrier (BBB), which serves as a physical barrier between the outside and inside of the brain. CLU-linked molecular pathways at the BBB’s interface are involved in the development and progression of AD.

### 4.4. Inflammatory Markers and Complement Proteins

**Figure 2:**
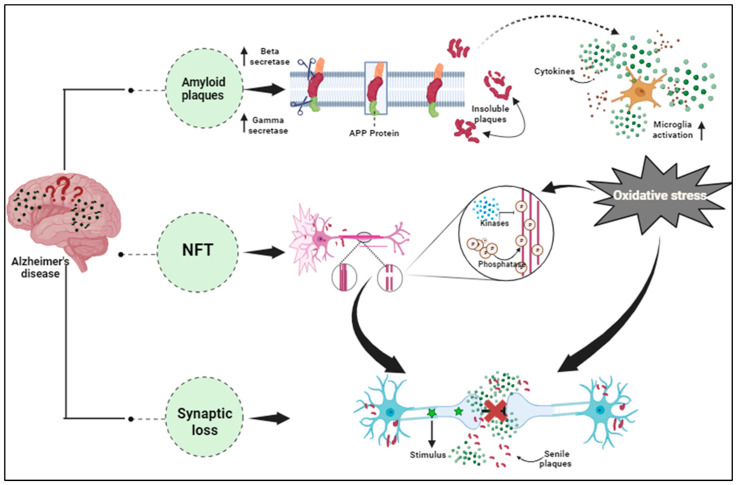
Pathological hallmarks of Alzheimer’s disease, including amyloid plaques, neurofibrillary tangles, and synaptic dysfunction.

It has been found that AD pathology even includes other factors excluding Aβ and NFTs, that are majorly involved in neuronal impairment. The increased expression of pro-inflammatory cytokines in the brain tissues and the blood samples of AD patients confirmed the association of inflammation in the advancement of various diseases including neurodegenerative diseases [118]. Angharad et al., 2019, worked on finding a plasma biomarker that aids early diagnosis, stratification, prediction of disease course, or monitoring response to therapy in AD [119]. Astrocytes, microglia, cytokines, and chemokines, which are part of the nervous system’s innate immune reaction known as neuroinflammation, are crucial in the pathogenesis of AD’s early stages Figure 2 [120]. The accumulated Aβ oligomers stimulate the microglia, initiating the release of pro-inflammatory mediators like glial fibrillary acidic protein (GFAP), neurotoxins, and free radicals [121]. These compounds elicit oxidative stress, thereby promoting inflammatory processes in neurons. The MAPK (Mitogen-activated protein kinase) pathway also promotes neurodegeneration by synchronizing with NF-*к*B (nuclear factor kappa-light-chain-enhancer of activated B cells) increasing pro-inflammatory cytokine production, leading to enhanced APP processing that hyper-phosphorylates the tau protein forming neurofibrillary tangles and eventually collapses the BBB (blood–brain barrier) [122]. In AD, exaggerated immune reactivity of p38 MAPK initiates cytokine production via direct phosphorylation of transcription factors and enhanced mRNA translation coding for pro-inflammatory cytokines [123]. Once the Aβ peptides accumulate during AD, an immune reaction in the brain initiates the activation and release of inflammatory markers such as TNF-*α*, IL-1, and IL-6, and the activation of specialized brain cells. Inflammatory markers such as tumor necrosis factor (TNF), interleukin-6 (IL-6), chitinase-3-like protein 1 (CHI3L1 or YKL-40), and acute phase C reactive protein (CRP) were found to have effects on the brains or peripheral regions of dementia patients [124]. The activated inflammatory cells further produce complement components within the CNS. In vitro, studies show direct activation of the classical pathways via Aβ1–42 and C1q binding. This C1q binds to Aβ and tau, possibly contributing to neuroinflammation and neurodegeneration [125]. Thus, this pro-inflammatory cascade can function as a channel promoting neuroinflammation and neurodegeneration [126].

### 4.5. Other Potential Proteomic Biomarkers for AD

Apart from the majorly prevalent biomarkers, alpha-Synuclein (α-SN), a pervasive 140 amino acid protein of 18–20 kDa molecular weight is found to be associated with AD. *SNCA* (Synuclein Alpha), encodes the α-SN, found profusely at the presynaptic terminal of neurons [127]. Early-onset AD (EOAD) patients have higher α-SN levels in their CSF, thereby showcasing a probable relation between *SNCA* gene polymorphisms and AD pathophysiology. α-SN possessing chaperone-like activity might play a role in the regulation of synaptic plasticity, neuronal differentiation, and up-regulation of dopamine release [128]. It is primarily associated with synuclein-associated pathologies such as Parkinson’s disease (PD) and dementia with Lewy bodies (DLB), where its misfolding and aggregation play a vital role in the neurodegeneration pathogenesis. DLB is neurodegenerative dementia characterized by the presence of abnormal aggregates of α-SN protein, termed Lewy bodies, in the brain [129]. The other associated biomarkers, like the *APOE*ε4 variant, significantly increase the risk of DLB. α-SN deposition or Lewy-related pathology is prominent in AD cases where patients experience visual hallucinations or symptoms that are often associated with Parkinson’s disease. In protein misfolding diseases, the accumulation of α-SN clogs the cellular machinery, disrupting the protein homeostasis network [130]. The early stage abnormal α-SN deposition at the presynaptic site in the brain shows its prominent presence in the early events of AD pathogenesis. α-SN directly interacts with Aβ and tau, promoting aggregation, thereby worsening the cognitive decline [131]. The abundance of α-SN at the center of Aβ plaques has been verified by immunolabeling using an α-SN antibody, confirming its influence in the formation of Aβ plaques. Increased levels of soluble α-SN monomeric and oligomeric proteins have been found in the temporal region of the AD brain [132]. NMR imaging has discovered the selective interaction of the monomeric form of tau with the C-terminal region of the α-SN monomer, thereby elevating its oligomerization and subsequent fibril formation [133]. Previous studies have been successful in establishing a relationship between α-SN and AD-associated genes such as *APP*, *PSEN1*, and *APOE*. Elevated α-SN levels in AD could facilitate Aβ oligomerization, tau phosphorylation, activation of kinases, dissociations of tau and tubulin, and tau aggregation. Furthermore, the association of α-SN with genetic factors, such as *APP*, *PSEN1*, and *APOE*, could accelerate AD pathology [134]. Apart from this, altered brain protein phosphorylation is a hallmark of AD, and phosphoproteomics offers an opportunity to identify the associated markers. Butterfield et al., 2019, reported two major types of kinases, serine/threonine- and tyrosine-kinases, that catalyzes phosphorylation of serine and threonine and tyrosine residues, respectively, and have a significant role in protein misfolding and aggregation. Along with kinases, protein phosphatases that remove protein-bound phosphate groups were also involved in the phosphorylation of proteins and, consequently, contributed to regulation of protein function [135]. Thus, more precise and accurate identification of AD-associated probable factors may help in developing novel therapies or modifying the existing treatments for AD patients.

## 5. Validation and Clinical Utility of Proteome-Based Biomarkers

The approach to be followed for validating protein biomarkers is the fit-for-purpose perspective. This approach is extensively used at numerous stages of biomarker assays and in their associated clinical applications. The procedure for quantifying biomarkers can be absolute or relative, based on the standard curve’s attributes, involving factors like parallelism, substituted matrix, and reference standards [136]. There is a need for carrying out apt method validation experiments on the following:

- Sample accumulation;
- Parallelism;
- Range finding;
- Relative precision and accuracy;
- Specificity and stability.

The validation of these factors is necessary for reaching the analytic benchmark, which is critical for a proposed study. To validate the factors mentioned earlier, two sampling methods were employed: stratified random sampling and cluster sampling. While considering the selection of method platform and validation, exploring the interactions between the target ligand/biomarkers and the concerned biotherapeutic, is imperative [137]. Using commercial diagnostic kits helps generate astonishing data. With a view to meeting the requirements for drug development, suitable validation experiments, kit comparison, et cetera, are carried out to protect the integrity of the drug development process [138]. Moreover, physicochemical techniques and multiplex assays are employed to augment the single-analyte ligand-binding assay for protein biomarkers.

### 5.1. Challenges in the Validation of Proteomic Biomarkers

The ultimate goal of developing potent proteomic biomarkers poses numerous challenges to be overcome. The accurate evaluation of the concerned biomarker is crucial for validating biomarkers. The evaluation of biomarkers must be performed in a well-organized manner in order to ensure the high efficiency of the whole process. An important hurdle before us is the extremely expensive and time-consuming procedure of biomarker evaluation [139]. The most widely accepted approach for handling this issue is molecular profiling. It is found to be a promising approach but the risk of generating overfitted models and biased results should be considered. In order to nullify these biases, it is essential to incorporate specimens from intervention or cohort trials. The skyrocketing expenses of biomarker validation processes have given birth to various novel study designs such as DNA pooling and sequential filtering [140]. To deal with the biases encountered with the data analysis procedures, the use of logistic regression for providing resistance to the challenge of model overfitting as well as nullifying model misspecification. Biomarker validation studies have to be highly extensive in order to minimize all kinds of biases. It is known that a hypothesis-driven biomarker panel is less likely to encounter the risk of biases. This minimized bias risk can be attributed to the small and prespecified panel size [141]. Moreover, it becomes relatively easy for the researchers to find the potential source of biases if they have knowledge about the biomarkers under consideration. The scenario is totally diametrical in the case of non-hypothesis-driven biomarkers. Model overfitting is also considered a significant statistical challenge while dealing with high-dimensional data. The statistical theory suggests that when the complexity/quality of the data becomes too large, as compared with the number of samples, the model will align itself with a specific data set rather than aligning itself with the mechanism that generated the data [142]. The application of this model for predicting disease in a new data set results in failure. This event is called overfitting. The conclusive answer for eliminating biases is accurate study design, sample collection processes, timing and assay procedures.

### 5.2. Clinical Studies and Diagnostic Accuracy of Proteomic Biomarkers

The two fundamental pillars of clinical research are biomarker prognosis and understanding the disease pathogenesis. The combination of proteomics with biomarkers has given rise to novel avenues to explore this field. Contemporarily, various clinical trials employing proteomics for evaluating and discovering biomarkers have been under process for many types of malignancies as well as other diseases [143]. The advent of technology comprising protein microarrays like RPPA enables the researcher to quantify multifarious endpoints in a high-throughput scenario. These endpoints involve expression levels of the fundamental proteins along with their activated forms that serve as crucial signaling points employed in survival, proliferation, and angiogenesis [144]. For instance, the main driving pathway for the development of serous ovarian cancers, caused due to somatic gain-of-function mutation on chromosome 3p in PI3KCA, is the PI3K pathway [145]. The molecular activation of PI3K leads to the initiation of a strong survival signal as it drives the Akt pathway. The molecular agents against PI3K and its protein effectors like Akt and mTOR are undergoing comprehensive clinical investigations in numerous types of malignancies. In order to evaluate the modulation of the AKT/mTOR/PI3K pathway, proteomic assays are being employed. Along with the PI3K pathway, there are numerous pathways downstream of the receptor tyrosine kinase (RTK) [146]. Recent investigations involve the development of therapeutics and analysis of proteomic endpoints. These clinical studies help us formulate the idea that proteomic biomarkers can prove to be a promising tool for diagnosing various diseases in the medical field. The protein profiles derived from liquid biopsies provide more extensive organ-specific data than DNA/RNA [147]. This approach helps to identify the origin of the tumor under study. According to the clinical studies conducted, it is evident that the employment of proteomic biomarkers in adjunction to nucleic acids significantly improves diagnostic accuracy, thereby enhancing the treatment of the disease.

### 5.3. Monitoring Disease Progression and Response to Treatment

Effective diagnosis and monitoring of the disease is crucial in patient care. Proteomics has unfolded monumental areas of research, which has significantly restructured the contemporary patient treatment paradigm. For instance, the interlinking of proteomics with cancer research has led to the development of therapeutically efficient biomarkers and drug targets. The fascinating aspect of proteomic research involves the revelation of novel biomarkers for better prognosis of various diseases. Proteomics allows us to perform simultaneous qualitative and quantitative protein profiling. For instance, during cancer progression, there are differences and changes observed in the protein profiles and protein distribution among the body tissues. These protein alterations can be investigated with the aid of proteomics. The leading technique for acquiring high-resolution spectra comprising mixed peptides is liquid chromatography–mass spectrometry (LC/MS) [148]. These techniques are also employed in the discovery of specific and sensitive novel biomarkers for numerous diseases, specifically cancer. Regulation of the treatment response is pivotal when it comes to providing treatment to patients. In diseases involving abnormal cell proliferation, like cancer, it becomes imperative to closely monitor the progression of the disease and evaluate the treatment response on the oncogenic cell [149]. This aspect is highly important for ascertaining an apt treatment plan for the patient, to avoid risking the patient’s life. With a view to investigating hepatitis B virus-associated hepatocellular carcinoma, an isobaric TMT-labeling proteomics approach has been adopted. This technique is carried out by collecting liver samples and adjacent healthy tissues from the patient’s body. In glioblastoma, proteomics analysis using LC/MS has unraveled the presence of pre-apoptotic competing mechanisms present in the synapse along with drug resistance [150]. The scope of proteomics to meticulously analyze the observed alterations in proteins gives rise to wide research avenues and better prognosis, hence aiding in better patient care.

### 5.4. Future Prospects and Integration with Other Diagnostic Approaches

The medical field is incessantly progressing with a view to improving perspectives toward patient care and treatment plans. Research in proteomics intends to enhance our potential for prognosis, diagnosis, and development of therapeutic drug targets and finally head toward the common end goal of medicine, which is personalized patient treatment. In the past few years, proteomics has proved its capability of transforming the medical field, but there is a need for more extensive research in order to extract all the benefits of using proteomic tools. Incorporation of proteomic tools in clinical laboratories requires comprehensive research to fill the void present in reproducibility and efficiency enhancement. The challenges involving consideration of biological, pre-analytical, and analytical variability should be tackled [151]. The requirement for skilled researchers and expensive equipment still proves to be a roadblock in the feasibility of using proteomic tools in practice. Hence, the main objective for proteomics will continue to be the identification of novel biomarkers for numerous diseases and the associated development of immunological and biochemical tests based on the data extrapolated using these biomarkers. The integration of these proteomic tools with other contemporary approaches allows us to generate profound data on the disease under investigation. Techniques like DNA microarrays, which are employed for disease profiling, LC/MS to identify post-translational alterations and point mutations, et cetera [152]. Protein chips have also been an emerging field to analyze the enzymatic activity of membranes and secreted proteomes associated with diseases.

## 6. Bioinformatics and Data Analysis in Proteomics for AD Biomarkers

Bioinformatic tools are commonly used for proteomic data analysis in Alzheimer’s disease research. Table 3 provides an overview of software and resources used for data preprocessing, statistical analysis, pathway analysis, and integration of multi-omics data.

The protein samples under consideration are allowed to go through three stages:

(i) Pre-mass spectrometry (MS) analysis;
(ii) Acquisition of MS data;
(iii) Post-MS Bioinformatics data processing.

These strategies enable researchers to achieve their objectives of protein quantification and identification. To cite a few strategies, one may adopt the bottom-up or top-down approach for full-length analysis of peptides and proteins correspondingly. Proteomics involves dealing with a highly diversified biochemical pool, which renders the top-down approach extremely challenging to practically conceive in the laboratory. For instance, the most exhaustive top-down-approach-based study identified more than 3000 protein isoforms derived from 1000 genes of human origin [153]. On the other hand, the bottom-up approach involves the analysis of peptides derived from completely digested full-length proteins. This significantly lowers the extreme biochemical diversification and enhances proteomic coverage to as much as 10,000 proteins [154]. Other various tasks bioinformatics tools carry out are:

(a) Protein Identification;
(b) Protein Quantification;
(c) Statistical Classification;
(d) Differential Expression;
(e) Network/Pathway Analysis;
(f) Integration of Multi-Omics;
(g) Hypotheses Generation.

### 6.1. Data Pre-Processing and Quality Control

Post-MS data analysis aims at extracting accurate and precise information from the data acquired from initial studies. It focuses on information extrapolation from raw MS data for identifying and quantifying proteins. MS spectral libraries and large protein databases equipped with computational software can be investigated to assign MS2 spectra to peptides [155]. This analysis has also led to the creation of varied search algorithms as well. One detrimental challenge of the protein-database investigation is the profound risk of false discovery. In order to eliminate it, the target-decoy strategy has been adopted to reduce the false-discover rate (FDR) of the concerned protein samples (for instance < 1%) [156]. After the identification of proteins is carried out, the samples are subjected to the process of protein quantification. The purpose of protein quantification is to evaluate the protein abundances in the samples under consideration. Further, the samples undergo differential expression (DE) and network analysis to formulate reproducible hypotheses. Quality analysis (QA) and automated quality control (QC) are carried out to detect biases associated with measurement, consistency verification and elimination of the probability of error propagation [157]. These tasks hold immense importance in data pre-processing and quality control. Data acquisition is rendered unfruitful if the techniques involved in data processing are inefficient. It is crucial to pay equal attention to the analysis strategies adopted to eliminate all possible biases.

### 6.2. Statistical Analysis and Identification of Significant Biomarkers

Evaluation of biomarkers is indeed an intricate process and plays a precious role as markers of diagnosis, severity of the disease, and risk. Although we have witnessed immense progress in the field of randomized control trials and the standardization of methodology, there is still a need for accomplishing milestones in the field of diagnostic and prognostic biomarkers. Several steps are essential for the identification of clinically significant biomarkers:

(a) They should be notably modified in the diseased patients as compared to the control group;
(b) Assessment of the diagnostic attributes of biomarkers;
(c) Comparison between the contemporary diagnostic tests and the concerned biomarkers;
(d) Evaluation of the quality of biomarkers, for instance, assessing rapidity, invasiveness, cost, technical challenges, et cetera.

For meticulously analyzing the clinical significance of biomarkers, it is inevitable to understand the physiological mechanisms of the synthesis, production and kinetic attributes of biomarkers.

#### 6.2.1. Decision Matrix

The key to assessing the diagnostic potential of biomarkers is through their specificity and sensitivity. Sensitivity deals with the capacity to diagnose a disease in the patient who truly suffers from that particular ailment. In contrast, specificity is the potential to completely rule out the possibility of the disease in patients who are completely healthy [158]. Computation of these indices requires information on the actual state of the patient and the results obtained from biomarker. We quantify the diagnostic accuracy of a test by the number of patients it has been able to correctly classify. The Youden index is employed to find out the difference between the actual performance of biomarker and the best possible performance it could have displayed.

#### 6.2.2. Likelihood Ratios

An alternative way to quantify the diagnostic value of a biomarker is the use of likelihood ratios (LHRs). These are basically the ratios of the likelihood of the conducted test result in the diseased as well as the non-diseased patient population. The striking feature of LHR is that they have the potential to quantify information about the presence of diseases observed in the diagnostic test.

#### 6.2.3. ROC Curve

The ROC curve is primarily the cumulative plotting of positive LHRs evaluated at numerous values of the diagnostic test. The ROC curve is a graphical representation of the data presented by the LHRs.

### 6.3. Pathway Analysis and Functional Annotation

In the complex web of bioinformatics, pathway analysis is employed to identify proteins associated with a pathway or construct a pathway de novo from the concerned proteins. It is highly useful while investigating the differential expression of the genes in disease and evaluating any omics dataset with a huge number of proteins. It is a type of data analysis whose objective is to elucidate activated pathways from the generated functional proteomic information. Pathway analysis enables the assemblage of a long list of proteins into concise pathway knowledge maps. This aids us in deciphering the molecular mechanisms associated with these modified proteins and their expressions. Different protein networks and biological pathway databases are accessible like KEGG, BioCarta, HPD, BioGRID, STRING, HAPPI, Reactome, and PAGED [159]. Functional annotation enables us to extract useful information from high-throughput proteomic data. Varied bioinformatics tools with different strategies have been developed for functional annotation of the data. This is the procedure of affixing biological knowledge to protein and gene sequences [160]. This is usually carried out with the help of a sequence alignment tool called BLAST for observing similarities and then annotating the concerned sequences on the basis of the acquired information. Functional annotation is carried out in three major steps:

(a) Recognizing the non-coding parts of protein chains;
(b) Carrying out gene prediction;
(c) Affixing biological information to this gathered data.

### 6.4. Integration of Multi-Omics Data for Systems-Level Understanding

The machine learning prediction algorithm is inefficient in the case of proteins due to the paucity of proteomics data. To solve this issue of insufficient data, the integration of transcriptome data helps to fill the voids of scarce proteomic data. It also predicts unrevealed proteomics profiles of novel cell lines. Different methods have been discovered to facilitate the interlinking of multi-omics data. Most of the contemporary interlinked methodologies are horizontal and unidirectional [161]. They tend to neglect the biological interconnections present between omics datasets and portray the hierarchical flow of a biological organization. For instance, a novel computational program known as TransPro has the potential to hierarchically amalgamate the multi-omics data of pharmacological-origin compound evaluation. It is primarily a deep learning model for the prediction of cell-particular proteomic profiles perturbed by unseen chemical substances [162]. Apart from integrating genomics, transcriptomics and proteomics, it can be further evolved to include phosphoproteomics and other omics data fields as well. The shortcomings of this deep learning technology are the difficulty faced while evaluating the enormous amount of generated data with the help of multi-omics readouts [163]. This algorithm makes computational prediction imperative. In a nutshell, these deep learning technologies have enabled us to incorporate and more specifically interlink to find associations between unlabeled, noisy, biased, and heterogenous omics data from varied origins.

## 7. Discussion

Recent groundbreaking research in the field of interlinking proteomics and AD has considerably widened our perspective on treatment intervention. The discovery of proteome-based biomarkers as a consequence of the substantial research on the MS-based proteomic profiling of the cerebrospinal fluid has been an important milestone. Novel dysregulated pathways, genes, and proteins have been characterized that influence AD and support the hypothesis of AD being a multifactorial pathological disease. The research involves the exploration of protein aggregation, which is a hallmark of AD, typically associated with Aβ and hyperphosphorylated tau-like proteins. Lutz et al., 2018, focused on identifying a aggregated proteome that could provide insight into the underlying mechanisms of AD development and progression. MS techniques coupled with plaque and NFT isolation allow for the analysis of the aggregate proteome in human AD samples. LCM and detergent-insoluble fractionation techniques have been successfully applied to isolate amyloid plaques and NFTs directly from AD brain samples for MS analysis. These techniques have identified novel aggregate proteins including U1-snRNP, a member of the spliceosome necessary for RNA splicing [164]. To conquer the quest for efficient profiling of AD biofluids like CSF and plasma proteomes, various deep-learning platforms have also been developed. These platforms couple important tasks like TMT labeling, sample processing, comprehensive 2D LC fractionation, and high-resolution tandem MS [165]. Along with the issues of proteome coverage, the issue of the reproducibility of results continues to be a complex one to handle. The primary challenge in identifying protein biomarkers in bodily fluids is the limited presence of these proteins at low abundance levels. Research indicates that future advancements in mass spectrometry, particularly those with high sensitivity, may address this roadblock. In the context of post translational modification (PTM) analysis, immunohistochemistry has been a widely employed technique to study PTMs in Alzheimer’s disease (AD). Researchers have used nano-LC MS/MS and MALDI-TOF MS to investigate proteins associated with AD. Interestingly, their findings revealed that oxidized forms of transthyretin (TTR) were actually found in lower quantities in the cerebrospinal fluid (CSF) of AD patients, compared to healthy individuals (Popov et al., 2013) [166]. Research suggests that the mitochondrial changes in the serum, CSF, and cortex are highly responsible for AD as supported by sufficient experimental evidence [167]. As a result, it was concluded that the astrocytic mitochondria are also released into the CSF as a potent biomarker for evaluating brain integrity accompanying low mitochondrial quantity in the brain, hinting towards brain damage. Briefly, the emergence of proteomics in AD research has significantly given a boost to the research sphere. Proteome-based biomarkers have proven not only to be a novel but also an efficient option for effective prognosis and diagnosis of AD thereby improving the quality of treatment offered to the patients.

## 8. Limitations and Future Directions in AD Biomarker Research

The phenotypic changes occurring in the human body associated with the disease are considered as dawning attributes modulated by temporal and spatial dimensional components and their numerous interactions with each other at the various levels of a hierarchical biological system. The advent of proteomics in AD has led to the formulation of novel interventions, which has the power to revolutionize the medical perspective toward prognosis. One major challenge faced is the contamination of the sample quality in AD proteomics by various factors. These unwanted alterations can be attributed to age, ischemia, gender, postmortem interval (PMI) et cetera. This unavoidable influence of these factors on the modified proteomes is far greater than the whole proteome; this can be because of the highly dynamic and transient nature of the protein alterations [168]. These influences can be dealt with with the help of regression analyses of the control experiments on animals. AD proteomics also faces obstacles of sample size, which is highly sensitive to experimental and biological variations. Proteomic studies with relatively small sample sizes give rise to biased results, which are not reproducible. The human brain comprises close to 16,000 genes that possess the potential to yield millions of proteoforms related to alternative RNA splicing. Research indicates that a large number of tau and Aβ proteoforms are present in AD brains as a consequence of proteolytic processes [169]. Bottom-up proteomics data deals with an enormous amount of brain proteomes, it is a tedious task of mapping intact proteoforms as their digestion leads to data loss. Advanced MS techniques have been developed to examine the protein structure of protein complexes and purified proteins and will further be evolved to dissect structural modifications in AD at an international level. In addition, the incessant enhancement of sensitivity, affordability, and throughput in proteomics renders the whole process considerably productive.

## 9. Conclusions

The cellular constituents of the human brain are highly heterogenous as observed in transition or homeostasis states. This cellular heterogeneity is usually masked with the bulk analyses carried out. The rapid transformation in the research perspective of AD can be attributed to the revolution caused by the interlinking of proteomics in this field. It is important to highlight the fact that there is a bridge between proteomic data creation and unearthing the drivers for specific diseases. This can be attributed to the capacity of proteomic profiling to reveal only disease-associated information; in other words, profiling educates us with correlation and not with causation. Recent studies have identified numerous DE proteins in AD brains that have left us with the task of validating the associated mechanisms that contribute to the molecular modifications. In a nutshell, contemporary deep proteomic studies have profiled the brain and related biofluids at an accelerated pace, leading to the generation of enormous data. This data is further used to formulate relevant hypotheses for validation. It is crucial to keep in mind that AD, being a neurodegenerative disease, creates numerous issues such as biological and cellular pathways being blocked, especially at the end-stage of the disease. With the coupling of MS and non-MS strategies with proteomics, we will witness monumental research and advancements in the treatment intervention of AD.

## Figures and Tables

**Table 1:** Comprehensive list of probable proteins and biomarkers associated with AD along with their molecular functions, cellular localization, and association with disease progression [31].

Biomarker	Location	Molecular Function	Role in AD
Amyloids	Arterial wall in brain	Helps in hormone release and plays a role in forming melanin, which helps protect the skin from sun damage.	Induces mitochondrial oxidative stress
p-Tau	Neurons, somato-dendritic compartments	Stabilizes neuronal microtubules and promotes axonal outgrowth	Accelerates the fibrillization of α-syn
Alpha-synuclein	In the axon terminals of presynaptic neurons	Role in neurotransmission at the synapse, calcium homeostasis, mitochondrial function, and gene regulation.	Induces the formation of Aβ oligomers; induces tau aggregation

**Table 2:** Antibodies used in Western blot analysis to identify amyloid β-protein (Aβ) with their corresponding Aβ recognition motifs and other motifs.

Recognition Motif	Nature of Antibody	Name of Antibody	Reference
Aβ_1–16_	Monoclonal	Ab9	[65]
Aβ_1–16_	Monoclonal	6C6	[66]
Aβ_1–17_	Monoclonal	6E10	[67]
Aβ_17–24_	Monoclonal	4G8	[68]
Aβ_31–40_	Monoclonal	2G3	[69]
Aβ_1–40_, C-terminal	Monoclonal	BA-27	[70]
Aβ_1–42_, C-terminal	Monoclonal	BC-05	[71]
Amyloid oligomers	Monoclonal	A8	[72]
Amyloid oligomers	Monoclonal	A11	[73]
Amyloid oligomers	Monoclonal	NU-4	[74]
Amyloid fibrils	Polyclonal	OC	[75]
Anti-amyloid beta precursor protein	Monoclonal	Y188	[76]
Anti-APP	Monoclonal	A8717	[77]
Anti-myelin basic protein	Monoclonal	MBP	[78]
Anti-kelch-like ECH-associated protein 1	Monoclonal	KEAP1	[79]

**Table 3:** Bioinformatic tools commonly used for proteomic data analysis in Alzheimer’s disease research. The table provides an overview of software and resources used for data preprocessing, statistical analysis, pathway analysis, and integration of multi-omics data.

Sr. No.	Purpose of the Tools	Tools and Softwares
1.	Identification of Data	Mascot
		SEQUEST
		OMSSA
		X!TANDEM
2.	Quantification of Data	MSQuant
		Progenesis
3.	Function and Localization	TMHMM
		Signal P
		PSORTb
4.	Pathway and Network Analysis	STRING
		Cytoscape
		KEGG
5.	Integration of Multi-Omics Data	iCluster
		TransPro
		PARADIGM
		LRAcluster
		PSDF
		BCC
		MDI

## Data Availability

Not applicable.

## References

[1] Long J.M., Holtzman D.M. (2019). Alzheimer Disease: An Update on Pathobiology and Treatment Strategies. Cell.

[2] Dubois B., Feldman H.H., Jacova C., Cummings J.L., Dekosky S.T., Barberger-Gateau P., Delacourte A., Frisoni G., Fox N.C., Galasko D. (2010). Revising the definition of Alzheimer’s disease: A new lexicon. Lancet Neurol..

[3] Masters C.L., Simms G., Weinman N.A., Multhaup G., McDonald B.L., Beyreuther K. (1985). Amyloid plaque core protein in Alzheimer disease and Down syndrome. Proc. Natl. Acad. Sci. USA.

[4] Alonso A.d.C., ElAkkad E., Gong C., Liu F., Tanaka T., Kudo T., Tatebayashi Y., Pei J., Wang J., Khatoon S. (2013). Inge Grundke-Iqbal, Ph.D. (1937–2012): The discoverer of the abnormal hyperphosphorylation of tau in Alzheimer’s disease. J. Mol. Neurosci..

[5] Roberson E.D., Halabisky B., Yoo J.W., Yao J., Chin J., Yan F., Wu T., Hamto P., Devidze N., Yu G.Q. (2011). Amyloid-β/Fyn-induced synaptic, network and cognitive impairments depend on tau levels in multiple mouse models of Alzheimer’s disease. J. Neurosci..

[6] Jack C.R., Bennett D.A., Blennow K., Carrillo M.C., Dunn B., Haeberlein S.B., Holtzman D.M., Jagust W., Jessen F., Karlawish J. (2018). NIA-AA Research Framework: Toward a biological definition of Alzheimer’s disease. Alzheimer’s Dement..

[7] Xu J., Chatterjee M., Baguley T.D., Brouillette J., Kurup P., Ghosh D., Kanyo J., Zhang Y., Seyb K., Ononenyi C. (2014). Inhibitor of the tyrosine phosphatase STEP reverses cognitive deficits in a mouse model of Alzheimer’s disease. PLoS Biol..

[8] Basir R., Hasballah K., Jabbarzare M., Gam L.H., Abdul Majid A.M., Yam M.F., Moklas M.A., Othman F., Che Norma M.T., Zalinah A. (2012). Modulation of interleukin-18 release produced positive outcomes on parasitaemia development and cytokines production during malaria in mice. Trop. Biomed..

[9] Srinivas P.R., Verma M., Zhao Y., Srivastava S. (2002). Proteomics for cancer biomarker discovery. Clin. Chem..

[10] Amiri-Dashatan N., Koushki M., Abbaszadeh H.A., Rostami-Nejad M., Rezaei-Tavirani M. (2018). Proteomics Applications in Health: Biomarker and Drug Discovery and Food Industry. Iran. J. Pharm. Res..

[11] Eyjolfsdottir H., Eriksdotter M., Linderoth B., Lind G., Juliusson B., Kusk P., Almkvist O., Andreasen N., Blennow K., Ferreira D. (2016). Targeted delivery of nerve growth factor to the cholinergic basal forebrain of Alzheimer’s disease patients: Application of a second-generation encapsulated cell biodelivery device. Alzheimer’s Res. Ther..

[12] Mirmosayyeb O., Tanhaei A., Sohrabi H.R., Martins R.N., Tanhaei M., Najafi M.A., Safaei A., Meamar R. (2017). Possible Role of Common Spices as a Preventive and Therapeutic Agent for Alzheimer’s Disease. Int. J. Prev. Med..

[13] Zali H., Zamanian-Azodi M., Rezaei Tavirani M., Akbar-Zadeh Baghban A. (2015). Protein Drug Targets of Lavandula angustifolia on treatment of Rat Alzheimer’s Disease. Iran. J. Pharm. Res..

[14] Karbalaei R., Allahyari M., Rezaei-Tavirani M., Asadzadeh-Aghdaei H., Zali M.R. (2018). Protein-protein interaction analysis of Alzheimer’s disease and NAFLD based on systems biology methods unhide common ancestor pathways. Gastroenterol. Hepatol. Bed Bench.

[15] Lei P., Ayton S., Bush A.I. (2021). The essential elements of Alzheimer’s disease. J. Biol. Chem..

[16] Hardy J., Allsop D. (1991). Amyloid deposition as the central event in the aetiology of Alzheimer’s disease. Trends Pharmacol. Sci..

[17] Scheltens P., Blennow K., Breteler M.M., de Strooper B., Frisoni G.B., Salloway S., Van der Flier W.M. (2016). Alzheimer’s disease. Lancet.

[18] Golembiewski J.A., Zeisel J. (2022). Salutogenic Approaches to Dementia Care. The Handbook of Salutogenesis.

[19] Goate A., Hardy J. (2012). Twenty years of Alzheimer’s disease-causing mutations. J. Neurochem..

[20] Xia D., Watanabe H., Wu B., Lee S.H., Li Y., Tsvetkov E., Bolshakov V.Y., Shen J., Kelleher R.J. (2015). Presenilin-1 knockin mice reveal loss-of-function mechanism for familial Alzheimer’s disease. Neuron.

[21] Eftekharzadeh B., Daigle J.G., Kapinos L.E., Coyne A., Schiantarelli J., Carlomagno Y., Cook C., Miller S.J., Dujardin S., Amaral A.S. (2018). Tau Protein Disrupts Nucleocytoplasmic Transport in Alzheimer’s Disease. Neuron.

[22] Glenner G.G., Wong C.W. (1984). Alzheimer’s disease: Initial report of the purification and characterization of a novel cerebrovascular amyloid protein. Biochem. Biophys. Res. Commun..

[23] Masters C.L., Multhaup G., Simms G., Pottgiesser J., Martins R.N., Beyreuther K. (1985). Neuronal origin of a cerebral amyloid: Neurofibrillary tangles of Alzheimer’s disease contain the same protein as the amyloid of plaque cores and blood vessels. EMBO J..

[24] Goldgaber D., Lerman M.I., McBride O.W., Saffiotti U., Gajdusek D.C. (1987). Characterization and chromosomal localization of a cDNA encoding brain amyloid of Alzheimer’s disease. Science.

[25] Rogaev E.I., Sherrington R., Rogaeva E.A., Levesque G., Ikeda M., Liang Y., Chi H., Lin C., Holman K., Tsuda T. (1995). Familial Alzheimer’s disease in kindreds with missense mutations in a gene on chromosome 1 related to the Alzheimer’s disease type 3 gene. Nature.

[26] Wolfe M.S., Xia W., Ostaszewski B.L., Diehl T.S., Kimberly W.T., Selkoe D.J. (1999). Two transmembrane aspartates in presenilin-1 required for presenilin endoproteolysis and gamma-secretase activity. Nature.

[27] Selkoe D.J., Hardy J. (2016). The amyloid hypothesis of Alzheimer’s disease at 25 years. EMBO Mol. Med..

[28] Arnsten A.F.T., Datta D., Del Tredici K., Braak H. (2021). Hypothesis: Tau pathology is an initiating factor in sporadic Alzheimer’s disease. Alzheimer’s Dement..

[29] Ballatore C., Lee V.M., Trojanowski J.Q. (2007). Tau-mediated neurodegeneration in Alzheimer’s disease and related disorders. Nat. Rev. Neurosci..

[30] Roberson E.D., Scearce-Levie K., Palop J.J., Yan F., Cheng I.H., Wu T., Gerstein H., Yu G.Q., Mucke L. (2007). Reducing endogenous tau ameliorates amyloid beta-induced deficits in an Alzheimer’s disease mouse model. Science.

[31] Kumar D., Aditi S., Lalit S. (2020). A Comprehensive Review of Alzheimer’s Association with Related Proteins: Pathological Role and Therapeutic Significance. Curr. Neuropharmacol..

[32] Hodes R.J., Buckholtz N. (2016). Accelerating Medicines Partnership: Alzheimer’s Disease (AMP-AD) Knowledge Portal Aids Alzheimer’s Drug Discovery through Open Data Sharing. Expert Opin. Ther. Targets.

[33] Bai B., Wang X., Li Y., Chen P.C., Yu K., Dey K.K., Yarbro J.M., Han X., Lutz B.M., Rao S. (2020). Deep Multilayer Brain Proteomics Identifies Molecular Networks in Alzheimer’s Disease Progression. Neuron.

[34] Tu L., Lv X., Fan Z., Zhang M., Wang H., Yu X. (2020). Association of Odor Identification Ability with Amyloid-β and Tau Burden: A Systematic Review and Meta-Analysis. Front. Neurosci..

[35] Teunissen C.E., Petzold A., Bennett J.L., Berven F.S., Brundin L., Comabella M., Franciotta D., Frederiksen J.L., Fleming J.O., Furlan R. (2009). A consensus protocol for the standardization of cerebrospinal fluid collection and biobanking. Neurology.

[36] Verwey N.A., van der Flier W.M., Blennow K., Clark C., Sokolow S., De Deyn P.P., Galasko D., Hampel H., Hartmann T., Kapaki E. (2009). A worldwide multicentre comparison of assays for cerebrospinal fluid biomarkers in Alzheimer’s disease. Ann. Clin. Biochem..

[37] Garfin D.E. (1990). One-dimensional gel electrophoresis. Methods in Enzymology.

[38] Lee P.Y., Saraygord-Afshari N., Low T.Y. (2020). The evolution of two-dimensional gel electrophoresis-from proteomics to emerging alternative applications. J. Chromatogr. A.

[39] Shevchenko G., Konzer A., Musunuri S., Bergquist J. (2015). Neuroproteomics tools in clinical practice. Biochim. Biophys. Acta (BBA)—Proteins Proteom..

[40] Friedman D.B., Hoving S., Westermeier R. (2009). Isoelectric focusing and two-dimensional gel electrophoresis. Methods Enzymol..

[41] Santoni V., Molloy M., Rabilloud T. (2000). Membrane proteins and proteomics: Un amour impossible?. Electrophor. Int. J..

[42] Rabilloud T. (1998). Use of thiourea to increase the solubility of membrane proteins in two-dimensional electrophoresis. Electrophoresis.

[43] Yan J.X., Kett W.C., Herbert B.R., Gooley A.A., Packer N.H., Williams K.L. (1998). Identification and quantitation of cysteine in proteins separated by gel electrophoresis. J. Chromatogr. A.

[44] Görg A., Klaus A., Lück C., Weiland F., Weiss W. (2007). Two-dimensional electrophoresis with immobilized pH gradients for proteome analysis. A Lab. Man..

[45] Craft G.E., Chen A., Nairn A.C. (2013). Recent advances in quantitative neuroproteomics. Methods.

[46] Lull M.E., Erwin M.S., Morgan D., Roberts D.C., Vrana K.E., Freeman W.M. (2009). Persistent proteomic alterations in the medial prefrontal cortex with abstinence from cocaine self-administration. Proteom. Clin. Appl..

[47] Naseri N.N., Ergel B., Kharel P., Na Y., Huang Q., Huang R., Dolzhanskaya N., Burré J., Velinov M.T., Sharma M. (2020). Aggregation of mutant cysteine string protein-α via Fe–S cluster binding is mitigated by iron chelators. Nat. Struct. Mol. Biol..

[48] Freeman W.M., Hemby S.E. (2004). Proteomics for protein expression profiling in neuroscience. Neurochem. Res..

[49] Baggerman G., Vierstraete E., De Loof A., Schoofs L. (2005). Gel-based versus gel-free proteomics: A review. Comb. Chem. High Throughput Screen..

[50] Thiede B., Höhenwarter W., Krah A., Mattow J., Schmid M., Schmidt F., Jungblut P.R. (2005). Peptide mass fingerprinting. Methods.

[51] Háda V., Bagdi A., Bihari Z., Timári S.B., Fizil Á., Szántay C. (2018). Recent advancements, challenges, and practical considerations in the mass spectrometry-based analytics of protein biotherapeutics: A viewpoint from the biosimilar industry. J. Pharm. Biomed. Anal..

[52] Stump M.J., Fleming R.C., Gong W.H., Jaber A.J., Jones J.J., Surber C.W., Wilkins C.L. (2002). Matrix-assisted laser desorption mass spectrometry. Appl. Spectrosc. Rev..

[53] Domon B., Aebersold R. (2006). Mass spectrometry and protein analysis. Science.

[54] Blackstock W.P., Weir M.P. (1999). Proteomics: Quantitative and physical mapping of cellular proteins. Trends Biotechnol..

[55] Hernandez P., Müller M., Appel R.D. (2006). Automated protein identification by tandem mass spectrometry: Issues and strategies. Mass Spectrom. Rev..

[56] Villar-Garea A., Griese M., Imhof A. (2007). Biomarker discovery from body fluids using mass spectrometry. J. Chromatogr..

[57] Palubeckaitė I. (2018). Analysis of Three-Dimensional Cell Cultures Using Mass Spectrometry Imaging.

[58] Grela A., Turek A., Piekoszewski W. (2012). Application of matrix-assisted laser desorption/ionization time-of-flight mass spectrometry (MALDI-TOF MS) in Alzheimer’s disease. Clin. Chem. Lab. Med..

[59] Korolainen M. (2006). Proteomic Analysis of Post-Translationally Modified Proteins in Alzheimer’s Disease (Alzheimerin Taudin Post-Translationaalisesti Muunneltujen Proteiinien Kartoittaminen Proteomiikan Avulla).

[60] Sobek J., Bartscherer K., Jacob A., Hoheisel J.D., Angenendt P. (2006). Microarray technology as a universal tool for high-throughput analysis of biological systems. Comb. Chem. High Throughput Screen..

[61] Ginsberg S.D., Hemby S.E., Lee V.M., Eberwine J.H., Trojanowski J.Q. (2000). Expression profile of transcripts in Alzheimer’s disease tangle-bearing CA1 neurons. Ann. Neurol..

[62] Housley W.J., Pitt D., Hafler D.A. (2015). Biomarkers in multiple sclerosis. Clin. Immunol..

[63] Ho L., Fivecoat H., Wang J., Pasinetti G.M. (2010). Alzheimer’s disease biomarker discovery in symptomatic and asymptomatic patients: Experimental approaches and future clinical applications. Exp. Gerontol..

[64] Jankowsky J.L., Zheng H. (2017). Practical considerations for choosing a mouse model of Alzheimer’s disease. Mol. Neurodegener..

[65] Levites Y., Das P., Price R.W., Rochette M.J., Kostura L.A., McGowan E.M., Murphy M.P., Golde T.E. (2006). Anti-Aβ42- and anti-Aβ40-specific mAbs attenuate amyloid deposition in an Alzheimer disease mouse model. J. Clin. Investig..

[66] Seubert P., Oltersdorf T., Lee M.G., Barbour R., Blomquist C., Davis D.L., Bryant K., Fritz L.C., Galasko D., Thal L.J. (1993). Secretion of β-amyloid precursor protein cleaved at the amino terminus of the β-amyloid peptide. Nature.

[67] Deng J., Hou H., Giunta B., Mori T., Wang Y.J., Fernandez F., Weggen S., Araki W., Obregon D., Tan J. (2012). Autoreactive-Aβ antibodies promote APP β-secretase processing. J. Neurochem..

[68] Baghallab I., Reyes-Ruiz J.M., Abulnaja K., Huwait E., Glabe C. (2018). Epitomic characterization of the specificity of the anti-amyloid Aβ monoclonal antibodies 6E10 and 4G8. J. Alzheimer’s Dis..

[69] Walsh D.M., Hartley D.M., Condron M.M., Selkoe D.J., Teplow D.B. (2001). In vitro studies of amyloid β-protein fibril assembly and toxicity provide clues to the aetiology of Flemish variant (Ala692→ Gly) Alzheimer’s disease. Biochem. J..

[70] Borchelt D.R., Thinakaran G., Eckman C.B., Lee M.K., Davenport F., Ratovitsky T., Prada C.M., Kim G., Seekins S., Yager D. (1996). Familial Alzheimer’s disease–linked presenilin 1 variants elevate Aβ1–42/1–40 ratio in vitro and in vivo. Neuron.

[71] Kawarabayashi T., Younkin L.H., Saido T.C., Shoji M., Ashe K.H., Younkin S.G. (2001). Age-dependent changes in brain, CSF, and plasma amyloid β protein in the Tg2576 transgenic mouse model of Alzheimer’s disease. J. Neurosci..

[72] Zhang Y., He J.S., Wang X., Wang J., Bao F.X., Pang S.Y., Yin F., Hu H.G., Peng X.L., Sun W.M. (2011). Administration of Amyloid-β 42 Oligomer-Specific Monoclonal Antibody Improved Memory Performance in SAMP8 Mice. J. Alzheimer’s Dis..

[73] Kayed R., Canto I., Breydo L., Rasool S., Lukacsovich T., Wu J., Albay R., Pensalfini A., Yeung S., Head E. (2010). Conformation dependent monoclonal antibodies distinguish different replicating strains or conformers of prefibrillar Aβ oligomers. Mol. Neurodegener..

[74] Lambert M.P., Velasco P.T., Chang L., Viola K.L., Fernandez S., Lacor P.N., Khuon D., Gong Y., Bigio E.H., Shaw P. (2007). Monoclonal antibodies that target pathological assemblies of Aβ. J. Neurochem..

[75] Isas J.M., Luibl V., Johnson L.V., Kayed R., Wetzel R., Glabe C.G., Langen R., Chen J. (2010). Soluble and mature amyloid fibrils in drusen deposits. Investig. Ophthalmol. Vis. Sci..

[76] Chebli J., Rahmati M., Lashley T., Edeman B., Oldfors A., Zetterberg H., Abramsson A. (2021). The localization of amyloid precursor protein to ependymal cilia in vertebrates and its role in ciliogenesis and brain development in zebrafish. Sci. Rep..

[77] García-Ayllón M.S., Lopez-Font I., Boix C.P., Fortea J., Sánchez-Valle R., Lleó A., Molinuevo J.L., Zetterberg H., Blennow K., Sáez-Valero J. (2017). C-terminal fragments of the amyloid precursor protein in cerebrospinal fluid as potential biomarkers for Alzheimer disease. Sci. Rep..

[78] Jingwu Z., Vandenbark A.A., Jacobs M.P., Offner H., Raus J.C. (1989). Murine monoclonal anti-myelin basic protein (MBP) antibodies inhibit proliferation and cytotoxicity of MBP-specific human T cell clones. J. Neuroimmunol..

[79] Kang C., Jeong S., Kim J., Ju S., Im E., Heo G., Park S., Yoo J.W., Lee J., Yoon I.S. (2023). N-Acetylserotonin is an oxidation-responsive activator of Nrf2 ameliorating colitis in rats. J. Pineal Res..

[80] Pryor N.E., Moss M.A., Hestekin C.N. (2012). Unraveling the early events of amyloid-β protein (Aβ) aggregation: Techniques for the determination of Aβ aggregate size. Int. J. Mol. Sci..

[81] Puig Gomà-Camps E. (2019). Structural Characterization of Amyloid Beta Oligomers with Functional Links Associated to Alzheimer’s Disease. https://dialnet.unirioja.es/servlet/tesis?codigo=250722.

[82] Miyoshi E., Bilousova T., Melnik M., Fakhrutdinov D., Poon W.W., Vinters H.V., Miller C.A., Corrada M., Kawas C., Bohannan R. (2021). Exosomal tau with seeding activity is released from Alzheimer’s disease synapses, and seeding potential is associated with amyloid beta. Lab. Investig..

[83] Ying Z., Xin W., Jin-Sheng H., Fu-Xiang B., Wei-Min S., Xin-Xian D., Xiao-Bo W., Yi-Qin L., Xian-Xian Z., Hong-Gang H. (2009). Preparation and characterization of a monoclonal antibody with high affinity for soluble Aβ oligomers. Hybridoma.

[84] Vaillant-Beuchot L., Mary A., Pardossi-Piquard R., Bourgeois A., Lauritzen I., Eysert F., Kinoshita P.F., Cazareth J., Badot C., Fragaki K. (2021). Accumulation of amyloid precursor protein C-terminal fragments triggers mitochondrial structure, function, and mitophagy defects in Alzheimer’s disease models and human brains. Acta Neuropathol..

[85] Yang Y.W., Hsu K.C., Wei C.Y., Tzeng R.C., Chiu P.Y. (2021). Operational Determination of Subjective Cognitive Decline, Mild Cognitive Impairment, and Dementia Using Sum of Boxes of the Clinical Dementia Rating Scale. Front. Aging Neurosci..

[86] Pham T.K., Buczek W.A., Mead R.J., Shaw P.J., Collins M.O. (2021). Proteomic approaches to study cysteine oxidation: Applications in neurodegenerative diseases. Front. Mol. Neurosci..

[87] García-Santamarina S., Boronat S., Domènech A., Ayté J., Molina H., Hidalgo E. (2014). Monitoring in vivo reversible cysteine oxidation in proteins using ICAT and mass spectrometry. Nat. Protoc..

[88] Martin B., Brenneman R., Becker K.G., Gucek M., Cole R.N., Maudsley S. (2008). iTRAQ analysis of complex proteome alterations in 3xTgAD Alzheimer’s mice: Understanding the interface between physiology and disease. PLoS ONE.

[89] Lundberg E., Borner G.H. (2019). Spatial proteomics: A powerful discovery tool for cell biology. Nat. Rev. Mol. Cell Biol..

[90] Dowling P., Gargan S., Murphy S., Zweyer M., Sabir H., Swandulla D., Ohlendieck K. (2021). The Dystrophin Node as Integrator of Cytoskeletal Organization, Lateral Force Transmission, Fiber Stability and Cellular Signaling in Skeletal Muscle. Proteomes.

[91] Baazaoui N., Iqbal K. (2022). Alzheimer’s Disease: Challenges and a Therapeutic Opportunity to Treat It with a Neurotrophic Compound. Biomolecules.

[92] Bloom G.S. (2014). Amyloid-β and Tau: The Trigger and Bullet in Alzheimer Disease Pathogenesis. JAMA Neurol..

[93] Hampel H., Hardy J., Blennow K., Chen C., Perry G., Kim S.H., Villemagne V.L., Aisen P., Vendruscolo M., Iwatsubo T. (2021). The Amyloid-β Pathway in Alzheimer’s Disease. Mol. Psychiatry.

[94] John A., Reddy P.H. (2021). Synaptic basis of Alzheimer’s disease: Focus on synaptic amyloid beta, P-tau and mitochondria. Ageing Res. Rev..

[95] Pereira J.B., Janelidze S., Ossenkoppele R., Kvartsberg H., Brinkmalm A., Mattsson-Carlgren N., Stomrud E., Smith R., Zetterberg H., Blennow K. (2021). Untangling the association of amyloid-β and tau with synaptic and axonal loss in Alzheimer’s disease. Brain.

[96] Li Y., Schindler S.E., Bollinger J.G., Ovod V., Mawuenyega K.G., Weiner M.W., Shaw L.M., Masters C.L., Fowler C.J., Trojanowski J.Q. (2022). Validation of plasma amyloid-β 42/40 for detecting Alzheimer disease amyloid plaques. Neurology.

[97] Hansson O., Lehmann S., Otto M., Zetterberg H., Lewczuk P. (2019). Advantages and disadvantages of the use of the CSF Amyloid β (Aβ) 42/40 ratio in the diagnosis of Alzheimer’s Disease. Alzheimer’s Res. Ther..

[98] Lue L.F., Guerra A., Walker D.G. (2017). Amyloid beta and tau as Alzheimer’s disease blood biomarkers: Promise from new technologies. Neurol. Ther..

[99] Raulin A.C., Doss S.V., Trottier Z.A., Ikezu T.C., Bu G., Liu C.C. (2022). ApoE in Alzheimer’s disease: Pathophysiology and therapeutic strategies. Mol. Neurodegener..

[100] Kanekiyo T., Bu G. (2014). The low-density lipoprotein receptor-related protein 1 and amyloid-β clearance in Alzheimer’s disease. Front. Aging Neurosci..

[101] Li Z., Shue F., Zhao N., Shinohara M., Bu G. (2020). APOE2: Protective mechanism and therapeutic implications for Alzheimer’s disease. Mol. Neurodegener..

[102] Kotze M.J., Lückhoff H.K., Brand T., Pretorius J., van Rensburg S.J. (2015). Apolipoprotein E ε-4 as a genetic determinant of Alzheimer’s disease heterogeneity. Degener. Neurol. Neuromuscul. Dis..

[103] Verghese P.B., Castellano J.M., Garai K., Wang Y., Jiang H., Shah A., Bu G., Frieden C., Holtzman D.M. (2013). ApoE influences amyloid-β (Aβ) clearance despite minimal apoE/Aβ association in physiological conditions. Proc. Natl. Acad. Sci. USA.

[104] Shi Y., Holtzman D.M. (2018). Interplay between innate immunity and Alzheimer disease: APOE and TREM2 in the spotlight. Nat. Rev. Immunol..

[105] Kurz C., Walker L., Rauchmann B.S., Perneczky R. (2022). Dysfunction of the blood-brain barrier in Alzheimer’s disease: Evidence from human studies. Neuropathol. Appl. Neurobiol..

[106] Milinkeviciute G., Green K.N. (2023). Clusterin/apolipoprotein J, its isoforms, and Alzheimer’s disease. Front. Aging Neurosci..

[107] Liu Y., Song J.H., Xu W., Hou X.H., Li J.Q., Yu J.T., Tan L., Chi S., Alzheimer’s Disease Neuroimaging Initiative (2021). The associations of cerebrospinal fluid ApoE and biomarkers of Alzheimer’s disease: Exploring interactions with sex. Front. Neurosci..

[108] Lennol M.P., Sánchez-Domínguez I., Cuchillo-Ibañez I., Camporesi E., Brinkmalm G., Alcolea D., Fortea J., Lleó A., Soria G., Aguado F. (2022). Apolipoprotein E imbalance in the cerebrospinal fluid of Alzheimer’s disease patients. Alzheimer’s Res. Ther..

[109] Bisht K., Sharma K., Tremblay M.È. (2018). Chronic stress as a risk factor for Alzheimer’s disease: Roles of microglia-mediated synaptic remodeling, inflammation, and oxidative stress. Neurobio Stress.

[110] Veitch D.P., Weiner M.W., Aisen P.S., Beckett L.A., Cairns N.J., Green R.C., Harvey D., Jack C.R., Jagust W., Morris J.C. (2019). Understanding disease progression and improving Alzheimer’s disease clinical trials: Recent highlights from the Alzheimer’s Disease Neuroimaging Initiative. Alzheimer’s Dement..

[111] Yuste-Checa P., Bracher A., Hartl F.U. (2022). The chaperone Clusterin in neurodegeneration-friend or foe?. Bioessays.

[112] Uddin M.S., Kabir M.T., Begum M.M., Islam M.S., Behl T., Ashraf G.M. (2021). Exploring the Role of CLU in the Pathogenesis of Alzheimer’s Disease. Neurotox. Res..

[113] Li K.W., Ganz A.B., Smit A.B. (2019). Proteomics of neurodegenerative diseases: Analysis of human post-mortem brain. J. Neurochem..

[114] Han S., Nho K., Lee Y. (2020). Alternative Splicing Regulation of an Alzheimer’s Risk Variant in CLU. Int. J. Mol. Sci..

[115] Fareed M.M., Qasmi M., Aziz S., Völker E., Förster C.Y., Shityakov S. (2022). The Role of Clusterin Transporter in the Pathogenesis of Alzheimer’s Disease at the Blood–Brain Barrier Interface: A Systematic Review. Biomolecules.

[116] Foster E.M., Dangla-Valls A., Lovestone S., Ribe E.M., Buckley N.J. (2019). Clusterin in Alzheimer’s Disease: Mechanisms, Genetics, and Lessons From Other Pathologies. Front. Neurosci..

[117] Yuste-Checa P., Trinkaus V.A., Riera-Tur I., Imamoglu R., Schaller T.F., Wang H., Dudanova I., Hipp M.S., Bracher A., Hartl F.U. (2021). The extracellular chaperone Clusterin enhances Tau aggregate seeding in a cellular model. Nat. Commun..

[118] De Oliveira J., Kucharska E., Garcez M.L., Rodrigues M.S., Quevedo J., Moreno-Gonzalez I., Budni J. (2021). Inflammatory cascade in Alzheimer’s disease pathogenesis: A review of experimental findings. Cells.

[119] Morgan A.R., Touchard S., Leckey C., O’Hagan C., Nevado-Holgado A.J., Barkhof F., Bertram L., Blin O., Bos I., Dobricic V. (2019). Inflammatory biomarkers in Alzheimer’s disease plasma. Alzheimer’s. Dement..

[120] Webers A., Heneka M.T., Gleeson P.A. (2020). The role of innate immune responses and neuroinflammation in amyloid accumulation and progression of Alzheimer’s disease. Immunol. Cell Biol..

[121] Rauf A., Badoni H., Abu-Izneid T., Olatunde A., Rahman M.M., Painuli S., Semwal P., Wilairatana P., Mubarak M.S. (2022). Neuroinflammatory Markers: Key Indicators in the Pathology of Neurodegenerative Diseases. Molecules.

[122] Chavarría C., Ivagnes R., Souza J.M. (2022). Extracellular Alpha-Synuclein: Mechanisms for Glial Cell Internalization and Activation. Biomolecules.

[123] Masenga S.K., Kabwe L.S., Chakulya M., Kirabo A. (2023). Mechanisms of Oxidative Stress in Metabolic Syndrome. Int. J. Mol. Sci..

[124] Rather M.A., Khan A., Alshahrani S., Rashid H., Qadri M., Rashid S., Alsaffar R.M., Kamal M.A., Rehman M.U. (2021). Inflammation and Alzheimer’s Disease: Mechanisms and Therapeutic Implications by Natural Products. Mediat. Inflamm..

[125] Shen X.N., Niu L.D., Wang Y.J., Cao X.P., Liu Q., Tan L., Zhang C., Yu J.T. (2019). Inflammatory markers in Alzheimer’s disease and mild cognitive impairment: A meta-analysis and systematic review of 170 studies. J. Neurol. Neurosurg. Psychiatry.

[126] Shah A., Kishore U., Shastri A. (2021). Complement System in Alzheimer’s Disease. Int. J. Mol. Sci..

[127] Agliardi C., Guerini F.R., Meloni M., Clerici M. (2022). Alpha-synuclein as a biomarker in Parkinson’s disease: Focus on neural derived extracelluar vesicles. Neural Reg. Res..

[128] Burré J., Sharma M., Südhof T.C. (2018). Cell Biology and Pathophysiology of α-Synuclein. Cold Spring Harb. Perspect. Med..

[129] Sengupta U., Guerrero-Muñoz M.J., Castillo-Carranza D.L., Lasagna-Reeves C.A., Gerson J.E., Paulucci-Holthauzen A.A., Krishnamurthy S., Farhed M., Jackson G.R., Kayed R. (2015). Pathological interface between oligomeric alpha-synuclein and tau in synucleinopathies. Biol. Psychiatry.

[130] Sweeney P., Park H., Baumann M., Dunlop J., Frydman J., Kopito R., McCampbell A., Leblanc G., Venkateswaran A., Nurmi A. (2017). Protein misfolding in neurodegenerative diseases: Implications and strategies. Transl. Neurodegener..

[131] Shim K.H., Kang M.J., Youn Y.C., An S.S.A., Kim S. (2022). Alpha-synuclein: A pathological factor with Aβ and tau and biomarker in Alzheimer’s disease. Alzheimer’s Res. Ther..

[132] Twohig D., Nielsen H.M. (2019). α-synuclein in the pathophysiology of Alzheimer’s disease. Mol. Neurodegener..

[133] Xu M.M., Ryan P., Rudrawar S., Quinn R.J., Zhang H.Y., Mellick G.D. (2020). Advances in the development of imaging probes and aggregation inhibitors for alpha-synuclein. Acta Pharmacol. Sin..

[134] Rodriguez-Vieitez E., Nielsen H.M. (2019). Associations Between APOE Variants, Tau and α-Synuclein. Adv. Exp. Med. Biol..

[135] Butterfield D.A. (2019). Phosphoproteomics of Alzheimer disease brain: Insights into altered brain protein regulation of critical neuronal functions and their contributions to subsequent cognitive loss. Biochim. Biophys. Acta Mol. Basis Dis..

[136] Iadecola C. (2004). Neurovascular regulation in the normal brain and in Alzheimer’s disease. Nat. Rev. Neurosci..

[137] Washburn M.P., Wolters D., Yates J.R. (2001). Large-scale analysis of the yeast proteome by multidimensional protein identification technology. Nat. Biotechnol..

[138] Toby T.K., Fornelli L., Kelleher N.L. (2016). Progress in Top-Down Proteomics and the Analysis of Proteoforms. Annu. Rev. Anal. Chem..

[139] Zhang Y., Fonslow B.R., Shan B., Baek M.C., Yates J.R. (2013). Protein analysis by shotgun/bottom-up proteomics. Chem. Rev..

[140] Nagaraj N., Wisniewski J.R., Geiger T., Cox J., Kircher M., Kelso J., Pääbo S., Mann M. (2011). Deep proteome and transcriptome mapping of a human cancer cell line. Mol. Syst. Biol..

[141] Xu P., Duong D.M., Peng J. (2009). Systematical Optimization of Reverse-Phase Chromatography for Shotgun Proteomics. J. Proteome Res..

[142] Cox J., Hein M.Y., Luber C.A., Paron I., Nagaraj N., Mann M. (2014). Accurate proteome-wide label-free quantification by delayed normalization and maximal peptide ratio extraction, termed MaxLFQ. Mol. Cell. Proteom..

[143] Meier F., Brunner A.D., Koch S., Koch H., Lubeck M., Krause M., Goedecke N., Decker J., Kosinski T., Park M.A. (2018). Online Parallel Accumulation-Serial Fragmentation (PASEF) with a Novel Trapped Ion Mobility Mass Spectrometer. Mol. Cell. Proteom..

[144] Ting L., Rad R., Gygi S.P., Haas W. (2011). MS3 eliminates ratio distortion in isobaric multiplexed quantitative proteomics. Nat. Methods.

[145] Akbani R., Becker K.F., Carragher N., Goldstein T., de Koning L., Korf U., Liotta L., Mills G.B., Nishizuka S.S., Pawlak M. (2014). Realizing the promise of reverse phase protein arrays for clinical, translational, and basic research: A workshop report: The RPPA (Reverse Phase Protein Array) society. Mol. Cell. Proteom..

[146] Liu P., Cheng H., Roberts T.M., Zhao J.J. (2009). Targeting the phosphoinositide 3-kinase pathway in cancer. Nat. Rev. Drug Discov..

[147] Gozal Y.M., Duong D.M., Gearing M., Cheng D., Hanfelt J.J., Funderburk C., Peng J., Lah J.J., Levey A.I. (2009). Proteomics analysis reveals novel components in the detergent-insoluble subproteome in Alzheimer’s disease. J. Proteome Res..

[148] Hales C.M., Seyfried N.T., Dammer E.B., Duong D., Yi H., Gearing M., Troncoso J.C., Mufson E.J., Thambisetty M., Levey A.I. (2014). U1 small nuclear ribonucleoproteins (snRNPs) aggregate in Alzheimer’s disease due to autosomal dominant genetic mutations and trisomy 21. Mol. Neurodegener..

[149] Raj T., Li Y.I., Wong G., Humphrey J., Wang M., Ramdhani S., Wang Y.C., Ng B., Gupta I., Haroutunian V. (2018). Integrative transcriptome analyses of the aging brain implicate altered splicing in Alzheimer’s disease susceptibility. Nat. Genet..

[150] Cheng Z., Shang Y., Xu X., Dong Z., Zhang Y., Du Z., Lu X., Zhang T. (2021). Presenilin 1 mutation likely contributes to U1 small nuclear RNA dysregulation and Alzheimer’s disease-like symptoms. Neurobiol. Aging.

[151] Cheng D., Hoogenraad C.C., Rush J., Ramm E., Schlager M.A., Duong D.M., Xu P., Wijayawardana S.R., Hanfelt J., Nakagawa T. (2006). Relative and absolute quantification of postsynaptic density proteome isolated from rat forebrain and cerebellum. Mol. Cell. Proteom..

[152] Butterfield D.A., Boyd-Kimball D., Castegna A. (2003). Proteomics in Alzheimer’s disease: Insights into potential mechanisms of neurodegeneration. J. Neurochem..

[153] Seyfried N.T., Dammer E.B., Swarup V., Nandakumar D., Duong D.M., Yin L., Deng Q., Nguyen T., Hales C.M., Wingo T. (2017). A Multi-network Approach Identifies Protein-Specific Co-expression in Asymptomatic and Symptomatic Alzheimer’s Disease. Cell Syst..

[154] Johnson E.C.B., Dammer E.B., Duong D.M., Yin L., Thambisetty M., Troncoso J.C., Lah J.J., Levey A.I., Seyfried N.T. (2018). Deep proteomic network analysis of Alzheimer’s disease brain reveals alterations in RNA binding proteins and RNA splicing associated with disease. Mol. Neurodegener..

[155] Wang H., Yang Y., Li Y., Bai B., Wang X., Tan H., Liu T., Beach T.G., Peng J., Wu Z. (2015). Systematic optimization of long gradient chromatography mass spectrometry for deep analysis of brain proteome. J. Proteome Res..

[156] Zhang Y., Sloan S.A., Clarke L.E., Caneda C., Plaza C.A., Blumenthal P.D., Vogel H., Steinberg G.K., Edwards M.S., Li G. (2016). Purification and Characterization of Progenitor and Mature Human Astrocytes Reveals Transcriptional and Functional Differences with Mouse. Neuron.

[157] Langfelder P., Horvath S. (2008). WGCNA: An R package for weighted correlation network analysis. BMC Bioinform..

[158] Wingo A.P., Liu Y., Gerasimov E.S., Gockley J., Logsdon B.A., Duong D.M., Dammer E.B., Robins C., Beach T.G., Reiman E.M. (2021). Integrating human brain proteomes with genome-wide association data implicates new proteins in Alzheimer’s disease pathogenesis. Nat. Genet..

[159] Esteve P., Rueda-Carrasco J., Inés Mateo M., Martin-Bermejo M.J., Draffin J., Pereyra G., Sandonís Á., Crespo I., Moreno I., Aso E. (2019). Elevated levels of Secreted-Frizzled-Related-Protein 1 contribute to Alzheimer’s disease pathogenesis. Nat. Neurosci..

[160] Liebmann T., Renier N., Bettayeb K., Greengard P., Tessier-Lavigne M., Flajolet M. (2016). Three-Dimensional Study of Alzheimer’s Disease Hallmarks Using the iDISCO Clearing Method. Cell Rep..

[161] Spilman P.R., Corset V., Gorostiza O., Poksay K.S., Galvan V., Zhang J., Rao R., Peters-Libeu C., Vincelette J., McGeehan A. (2016). Netrin-1 Interrupts Amyloid-beta Amplification, Increases sAbetaPPalpha in vitro and in vivo, and Improves Cognition in a Mouse Model of Alzheimer’s Disease. J. Alzheimer’s Dis..

[162] Wright J.W., Harding J.W. (2015). The Brain Hepatocyte Growth Factor/c-Met Receptor System: A New Target for the Treatment of Alzheimer’s Disease. J. Alzheimer’s Dis..

[163] Zheng H., Jia L., Liu C.C., Rong Z., Zhong L., Yang L., Chen X.F., Fryer J.D., Wang X., Zhang Y.W. (2017). TREM2 Promotes Microglial Survival by Activating Wnt/beta-Catenin Pathway. J. Neurosci..

[164] Lutz B.M., Peng J. (2018). Deep profiling of the aggregated proteome in Alzheimer’s disease: From pathology to disease mechanisms. Proteomes.

[165] Zhang D.F., Fan Y., Xu M., Wang G., Wang D., Li J., Kong L.L., Zhou H., Luo R., Bi R. (2019). Complement C7 is a novel risk gene for Alzheimer’s disease in Han Chinese. Natl. Sci. Rev..

[166] Popov I.A., Starodubtseva N.L., Indeikina M.I., Kostyukevich Y.I., Kononikhin A.S., Nikolaeva M.I., Kukaev E.N., Kozin S.A., Makarov A.A., Nikolaev E.N. (2013). Mass spectrometric identification of posttranslational modifications in transthyretin from human blood. Mol. Biol..

[167] Hong S., Beja-Glasser V.F., Nfonoyim B.M., Frouin A., Li S., Ramakrishnan S., Merry K.M., Shi Q., Rosenthal A., Barres B.A. (2016). Complement and microglia mediate early synapse loss in Alzheimer mouse models. Science.

[168] Morgan B.P. (2018). Complement in the pathogenesis of Alzheimer’s disease. Semin. Immunopathol..

[169] Litvinchuk A., Wan Y.W., Swartzlander D.B., Chen F., Cole A., Propson N.E., Wang Q., Zhang B., Liu Z., Zheng H. (2018). Complement C3aR Inactivation Attenuates Tau Pathology and Reverses an Immune Network Deregulated in Tauopathy Models and Alzheimer’s Disease. Neuron.

